# Decoding the tumor microenvironment and molecular mechanism: unraveling cervical cancer subpopulations and prognostic signatures through scRNA-Seq and bulk RNA-seq analyses

**DOI:** 10.3389/fimmu.2024.1351287

**Published:** 2024-02-28

**Authors:** Zhiheng Lin, Xinhan Li, Hengmei Shi, Renshuang Cao, Lijun Zhu, Chunxiao Dang, Yawen Sheng, Weisen Fan, Zhenghui Yang, Siyu Wu

**Affiliations:** ^1^ Shandong University of Traditional Chinese Medicine, Jinan, Shandong, China; ^2^ Department of Obstetrics and Gynecology, Women’s Hospital of Nanjing Medical University, Nanjing Maternity and Child Health Care Hospital, Nanjing, Jiangsu, China; ^3^ Wangjing Hospital of Chinese Academy of Chinese Medical Sciences, Beijing, China; ^4^ Longhua Hospital of Shanghai University of Traditional Chinese Medicine, Shanghai, China; ^5^ Zunyi Medical University, Zhuhai, Guangdong, China; ^6^ Department of Gynecology and Obstetrics, Qilu Hospital, Cheeloo College of Medicine, Shandong University, Qingdao, China

**Keywords:** cervical cancer, ScRNA-seq, bulk RNA-seq, PLP2+ Tumor EPCs, experiment validation

## Abstract

**Background:**

Cervical carcinoma (CC) represents a prevalent gynecological neoplasm, with a discernible rise in prevalence among younger cohorts observed in recent years. Nonetheless, the intrinsic cellular heterogeneity of CC remains inadequately investigated.

**Methods:**

We utilized single-cell RNA sequencing (scRNA-seq) transcriptomic analysis to scrutinize the tumor epithelial cells derived from four specimens of cervical carcinoma (CC) patients. This method enabled the identification of pivotal subpopulations of tumor epithelial cells and elucidation of their contributions to CC progression. Subsequently, we assessed the influence of associated molecules in bulk RNA sequencing (Bulk RNA-seq) cohorts and performed cellular experiments for validation purposes.

**Results:**

Through our analysis, we have discerned C3 PLP2+ Tumor Epithelial Progenitor Cells as a noteworthy subpopulation in cervical carcinoma (CC), exerting a pivotal influence on the differentiation and progression of CC. We have established an independent prognostic indicator—the PLP2+ Tumor EPCs score. By stratifying patients into high and low score groups based on the median score, we have observed that the high-score group exhibits diminished survival rates compared to the low-score group. The correlations observed between these groups and immune infiltration, enriched pathways, single-nucleotide polymorphisms (SNPs), drug sensitivity, among other factors, further underscore their impact on CC prognosis. Cellular experiments have validated the significant impact of ATF6 on the proliferation and migration of CC cell lines.

**Conclusion:**

This study enriches our comprehension of the determinants shaping the progression of CC, elevates cognizance of the tumor microenvironment in CC, and offers valuable insights for prospective CC therapies. These discoveries contribute to the refinement of CC diagnostics and the formulation of optimal therapeutic approaches.

## Introduction

Cervical carcinoma (CC) ranks as the fourth most prevalent malignancy and represents a leading contributor to global female cancer-related mortality (1). In both incidence and mortality profiles, CC consistently maintains a prominent status among the most prevalent cancer types worldwide in women, trailing behind only breast cancer, colorectal cancer, and lung cancer (2). Despite the substantial preventive potential provided by efficient screening and vaccination initiatives against CC, vaccination uptake remains notably deficient, with only a minority of women accessing comprehensive healthcare services (3). Consequently, the mortality rate for advanced-stage CC patients remains elevated, with a median survival period of a mere 16.8 months (4), and an average 5-year survival rate of 72% (5). Due to chemotherapy resistance, the current efficacy of chemotherapeutic agents is limited, exhibiting a response rate ranging from 29% to 63% (6). Standard care for CC presently encompasses radiotherapy, chemotherapy, or surgical resection. However, these approaches entail significant side effects, possess limited efficacy for advanced-stage diseases, and offer few treatment alternatives for cases of recurrence or metastasis (7). Although some treatments targeting advanced or recurrent CC, such as anti-angiogenesis and immunotherapy, exist, their response rates remain suboptimal (8). Given the low cure rates for advanced-stage diseases and the adverse effects associated with current therapies, there is an urgent need to provide new treatment options for CC patients.

To comprehend CC, it is imperative to initially comprehend the tumor microenvironment (TME) within which CC tumors arise and proliferate. The tumor microenvironment, consisting of diverse cellular components such as fibroblasts, endothelial cells (ECs), and immune cells, in conjunction with extracellular constituents including cytokines, hormones, extracellular matrix, and growth factors, constitutes an intricate network enveloping CC cells. The TME plays a pivotal role not only in the initiation, progression, and metastasis of CC but also profoundly influences therapeutic outcomes (9). Chemoresistance mediated by the TME is a result of intricate crosstalk between CC cells and their surrounding environment. In previous studies, investigations into the molecular mechanisms and chemoresistance of CC patients have predominantly focused on bulk genomic or transcriptomic analysis methods and *in situ* hybridization techniques (10). Consequently, research on chemoresistance mechanisms based on distinct characteristics of cell populations remains ambiguous.

Today, single-cell RNA sequencing (scRNA-seq) technology has emerged as a powerful tool for analyzing cell population spectra within tissues. This technique has been widely employed to elucidate complex subpopulations in organ tissues, such as the lungs (11), heart (12), and brain (13), as well as various cancers, including melanoma (14), ovarian (15), and colorectal (16) cancers (17). Single-cell sequencing stands as the latest tool for revealing tumor cell heterogeneity and the microenvironment. However, its application in clinical samples of CC remains limited.

In 2021, Hua et al. utilized scRNA-seq to investigate the intratumoral heterogeneity of CSCC based on tumor tissue and adjacent normal tissue from a single patient (18). While there are some comparative studies on TME changes before and after chemotherapy, relevant reports are concise and lack detailed exploration of scRNA-seq data. Currently, the use of scRNA-seq for mapping studies has been widely embraced by the scientific community (19).

Therefore, this study employed single-cell RNA sequencing (scRNA-seq) on CC samples to decipher the immunological microenvironment of CC. Unraveling the immune landscape of CC may offer novel insights into the treatment of advanced-stage CC, potentially expediting the eradication of cervical cancer. The paper extensively discusses and summarizes the functional roles and clinical relevance of tumor epithelial cell subpopulations during the progression of CC. This study provides a valuable resource and deeper insights into CC initiation and progression, which is helpful in refining CC diagnosis and for the design of optimal treatment strategies.

## Methods

### Data source

ScRNA-seq data were obtained from the GEO website (https://www.ncbi.nlm.nih.gov/geo/),GSE number was GSE171894. The number of patients is four. Each patient had a sample sequenced. The samples included: GSM5236544, GSM5236545, GSM5236546, GSM5236547. The bulk data was from the The Cancer Genome Atlas (TCGA) official website (https://portal.gdc.cancer.gov/).

### Processing of scRNA-seq data

The raw gene expression substrates were processed with the Seurat software package (version 4.3.0) (20). High-quality cells were obtained according to the following criteria: removed cells with extreme nFeature, nCount values; mitochondrial gene expression below 20% of the total number in one cell, and erythrocyte gene expression below 5% of the total number in one cell.Potential double cells was removed by using the DoubletFinder package. The samples were normalized to find the top 2000 highly variable genes, and their data were normalized (21, 22). Subsequently, we further analyzed the data by PCA and we used the method of harmony in order to remove the batch effect between samples. The top 30 significant principal components (PCs) were selected for uniform manifold approximation and projection (UMAP) downscaling and gene expression visualization (23, 24). Cell clusters were annotated by cell markers obtained from previous literature and according to the CellMarker database (http://xteam.xbio.top/CellMarker/). Subsequently, we also observed the proportion of different cell types (25–27).

### DEGs and GESA

Differentially Expressed Genes (DEGs) of per cell type were identified by performing the FindAllMarker function on the normalized expression data in the Seurat software package (28, 29), and genes expressed in more than 25% of the cells in clusters with a logFC value greater than 0.25 were selected.Genes with adjusted p-values <0.05 were considered statistically significant in KEGG and GO enrichment analysis. The ClusterProfiler software package (version 0.1.1) was used to enrich and analyze cluster-specific biomarker genes (30).

### Tumor cell and non-malignant epithelial cell differentiation based on inferred CNVs

To differentiate tumor cells and non-malignant epithelial cells, the initial copy number variation (CNV) signal was estimated for each region by using the inferCNV package (https://github.com/broadinstitute/inferCNV/wiki). Where NK/T cells were used as a reference, while we defined subpopulations with a high copy number variation profile as tumor epithelial cells.

### Determination of cell subpopulations

All tumor epithelial cells were extracted and renormalized to find the first 2000 highly variable genes, and their data were normalized. Subsequently, we further analyzed them by PCA and we used the method of harmony in order to remove the batch effect among samples. The expression of known typical marker genes for the respective cell types was annotated according to cell subtype. Cell subclusters with similar gene expression patterns were annotated to the same cell type and projected onto a 2D map by using the UMAP approach.

### Differential and enrichment analysis of cell subpopulations

We further used the “FindAllMarkers” function of the Wilcoxon rank-sum test to identify differential genes in each subpopulation of tumor epithelial cell subgroups and performed GO-BP enrichment analysis by using ClusterProfiler.

### Trajectory analysis

To further investigate CC tumorigenesis, trajectory analysis of tumor epithelial cell subpopulations was performed by using three software packages.

First, the algorithm of cytoTRACE was used to assess the cell stemness of each subpopulation of cells. Then, we further used the Monocle software toolkit to reconstruct cell differentiation trajectories, used DDRTree to downscale and to observe the development of the subpopulation cells under the newly created trajectories. Finally, we further analyzed the cell trajectories during tumor epithelial cell differentiation using the slingshot method, which was used to infer cell lineages and to estimate the cell expression level of each lineage over the pseudotime.

### Analysis of cell-cell interactions

To investigate the cell-cell interaction network between epithelial cell subpopulations and other microenvironmental cells, ligand-receptor pairs between ecotone cell subtypes and malignant cells were explored using the “CellChat” package (version 1.6.1) (31). We inferred cell-cell communication at the signaling pathway and receptor-ligand levels and explored how signaling pathways were coordinating across multiple cell types.

### Constructed novel immune-related features

To focus on the role of cervical cancer in predicting patient survival, we used key subgroups of cervical cancer marker genes, which were used to predict genetic signatures. We used univariate COX regression analysis based on the “survival” package to explore the correlation between the expression of these important genes and the overall survival (OS) of cervical cancer patients (32). LASSO regression analysis was performed by using the R package “survival” to find the more important prognostic genes. The risk score for each cervical cancer patient was calculated based on the expression level of each gene and the corresponding LASSO regression coefficient.Riskscore = Expression of gene 1 * coefficient 1 + Expression of gene 2 * coefficient 2 +… + Expression of gene n * coefficient n. And groups were divided based on the median, above the median was high score group and below the median was low score group (33, 34). We further did survival analysis to observe the prognosis of patients in different groups. The accuracy was assessed by plotting the subjects’ work characteristics (ROC) curves at 1, 3, and 5 years by using the timeROC software package (version 0.4.0) (35, 36). In addition, we further explored the relationship between modeled genes and risk scores and OS.

### Construction and validation of nomogram plots

Nomogram plot was constructed based on the risk score, M-stage, N-stage and etc.of the new characteristics to predict the 1-year, 3-year, and 5-year overall survival probabilities of cervical cancer patients in the TCGA cohort (37, 38). The predictive ability of the model was also evaluated by using the C-index score.

### Estimation of immune cell infiltration

The immune cell infiltration in each CESC sample of the TCGA dataset was estimated by the computational analysis tools CIBERSORT (http://cibersort.stanford.edu/) (39), ESTIMATE, and Xcell. Subsequently, we further observed the high or low level of immune cells in different groups under the CIBERSORT algorithm, and further observed their correlation with risk score, modeling genes, and OS. In addition, we also observed the high and low situation of Stromal Score, Immune Score,EATIMATE Score and TumorPurity in different groups.

### Differential and enrichment analysis of bulk data

We used the “DESeq2” package to analyze the differences between the high and low risk group, with a threshold of |logFC|>2 and a p-value of less than 0.05, and used the ClusterProfiler package to analyze the GO, KEGG, and GSEA enrichment of the differential genes (40–42).

### Somatic mutation analysis

The mutation data for somatic mutation analysis were obtained from the TCGA database, and we observed the mutation distribution of highly mutated genes and modeled genes. The tumor mutation load (TMB) of each tumor epithelial cell sample was calculated using the “maftools” software package, and the cervical cancer samples were classified into high and low TMB groups according to the median tumor mutation load (TMB). And Kaplan-Meier was used to observe the survival differences among different groups. In addition, we further observed the CNV profiles of the modeled genes.

### Immunotherapy effect in predicting chemotherapy response

We used the “pRRophetic” package (version 0.5) to infer the half-maximal inhibitory concentration (IC50) of chemotherapeutic drugs (43), and assessed the drug sensitivity of chemotherapeutic drugs in different groups (44, 45).

### Cell culture

SiHa and Hela cells came from the American type culture collection (ATCC). Both cell lines were cultured with DMEM medium (Gibco BRL, USA) supplemented with 10% fetal bovine serum (Gibco BRL, USA) in a 5% CO2 incubator at 37°C.

### Cell transfection and RT-qPCR

Two small interfering RNAs (siRNAs) targeting ATF6 genes and their corresponding negative controls (si-NC) were synthesized by Ribobio (Guangzhou, China), The transfection regimen was performed according to the protocol for Lipofectamine 3000(Invitrogen, USA).

Total RNA was extracted from cell lines using TRIzol reagent (15596018, Thermo) and the RNA concentration was standardized. Subsequently, cDNA was synthesized using PrimeScript™RT kit (R232-01, Vazyme). SYBR Green Kit (TaKaRa Biotechnology, Dalian, China) is used for real-time quantitative PCR (qRT-PCR). GAPDH was used as an internal reference. **Supplementary Table 1** contains sequences of primers and siRNAs.

### Cell counting

5×103 transfected cells were implanted in each of the 96-well plates (Corning, USA, 3599). After waiting for cell attachment, cells were treated with CCK-8 labeling reagent (A311-01, Vazyme) at 1, 2, 3, 4, and 5 days, respectively, and incubated for 2 hours away from light, and OD value was recorded.

### Wound healing

The transfected cells were cultured in a 6-well plate (Corning, USA, 3516). When the cell density reached about 95%, a sterile pipette with a volume of 200 μL was used to cut the cell layer along a straight line. Rinse gently with PBS to remove unattached cells and debris. Subsequently, the serum-free cell medium was replaced to maintain cell growth. The photos were taken at the same location at 0 and 48 hours respectively.

### Transwell

Cells (corning, USA, 3412-01) with or without Matrigel matrix (BD Biosciences, USA) were placed in 24-well plates for transwell experiments. 1×104 cells were placed in each upper chamber and cultured with 200 microliters of serum-free DMEM medium. A complete DMEM medium with 700 µl 10% serum was added to the lower chamber. Culture in the incubator for 36 hours, after the cells of the upper chamber penetrate the lower chamber, the chamber is cleaned, fixed, and stained.

## Results

### scRNA sequencing revealed the main cell types in the progress of CC

**Figure 1:**
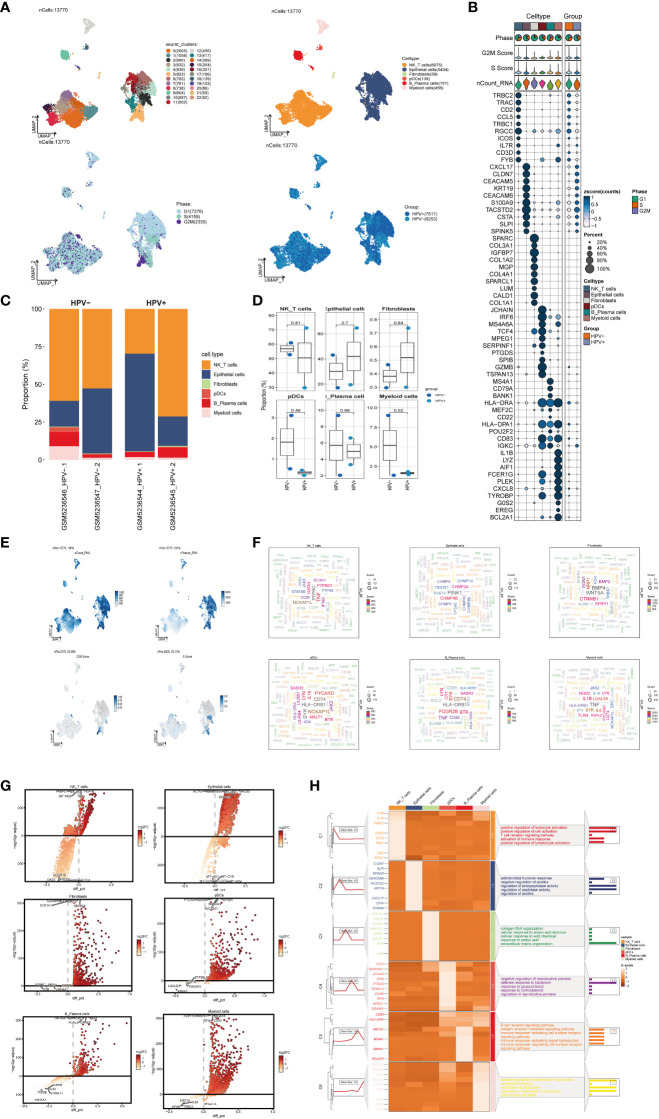
scRNA sequencing revealed major cell types during CC progression, **(A)** UMAP plot showed the 23 clusters of cells in cervical cancer patients and the number of cells in each cluster (top left); UMAP plot showed the six major cell types obtained by down clustering of cells in cervical cancer (top right); UMAP plot showed the distribution of phases in the six cell types (bottom left) and the infection of HPV (bottom right). Each point corresponded to a single cell colored according to cell cluster or cell type. **(B)** Bubble plot showed differential expression of Top10 maker genes in cervical cancer cells across cell types (NK/T cells, Epithelial cells, Fibroblasts, pDCs, B_Plasma cells, Myeloid cells). Bubble colors were based on normalized data and sizes indicated the percentage of genes expressed in the subpopulation. **(C)** Histograms depicted the percentage of the 6 major cell types in each of the 4 cervical cancer samples. **(D)** Box line plot depicted the percentage of the 6 cell types in the HPV+ group versus the HPV- group. The colors of the dots represented the HPV- and HPV+ groups, respectively. p-values corresponded to paired Wilcoxon tests. **(E)** UMAP plots visualized the relevant features of 6 cell types: nCount_RNA, nFeature_RNA, S.score, G2M.score. **(F)** Word cloud plots showed gene enrichment in the 6 cell types. The size of the letter indicated the number of genes and the color indicated the high or low enrichment score of the cell type. **(G)** Volcano plots demonstrated the expression of differential genes in the 6 cell types. **(H)** GO-BP enrichment analysis demonstrated the biological processes associated with the 6 cell types.

In order to obtain the main cell types in the progress of cervical cancer, we collected CC samples from 4 patients with cervical cancer to obtain scRNA-seq. After initial quality control and batch effect removal, a total of 13770 cells were retained. We clustered these 13,770 cells by dimensionality reduction, and the analysis revealed 23 unique tissue states (upper left of **Figure 1A**). Based on the typical tissue type-specific markers defined in the literature, we divided the cell clusters into six main cell types: NK_T cells(6975), Epithelial cells(5434), Fibroblasts(59), pDCs(139), B_Plasma cells(707) and Myeloid cells(456). The proportion of each cell type in different patients was very different, among which Epithelial cells were the most abundant structural cells, accounting for the largest proportion of all cells. UMAP diagram was used to show the phase situation (lower left in **Figure 1A**) and HPV infection (lower right in **Figure 1A**) in six cell types.

The expression of marker genes in six cell types were different, and the marker genes (top10) in each cell type were displayed by bubble diagram (**Figure 1B**). Bar charts were used to show the proportion of six cell types in HPV+ group and HPV- group. Although the proportion of cells in each sample was different, NK_T cells and Epithelial cells were the cell types with high proportion in all samples (**Figure 1C**). However, the results of box chart showed that there was no statistical difference in the proportion of these six cell types between groups (**Figure 1D**). UMAP diagrams were used to display nCount_RNA,nFeature_RNA, S. Score and G2m. Score of all cells (**Figure 1E**). The word cloud diagrams (**Figure 1F**) and volcano diagrams (**Figure 1G**) were used to describe the differential genes of these six cell types. The results of GO-BP enrichment analysis of six cell types were displayed by thermogram (**Figure 1H**). It could be seen that NK_T cells were related to biological processes such as positive regulation of leukocyte activation, positive regulation of cell activation, T cell receptor signaling pathway, etc. Epithelial cells were related to biological processes such as antimicrobial humoral response, negative regulation of anoikis, regulation of endopeptidase activity, etc. Fibroblasts were related to biological processes such as collagen fibril organization, cellular response to amino acid stimulus, cellular response to acid chemical, etc. PDCs were related to negative regulation of reproductive process, defense response to bacterium, response to glucocorticoid and other biological processes. B_Plasma cells were related to biological processes such as B cell receptor signaling pathway, antigen receptor−mediated signaling pathway, immune response−activating cell surface receptor signaling pathway and other biological processes. Myeloid cells were related to positive regulation of interleukin−6 production, neutrophil activation, interleukin−6 production and other biological processes.

### Visualization of cervical cancer tumor epithelial cell subgroups

**Figure 2:**
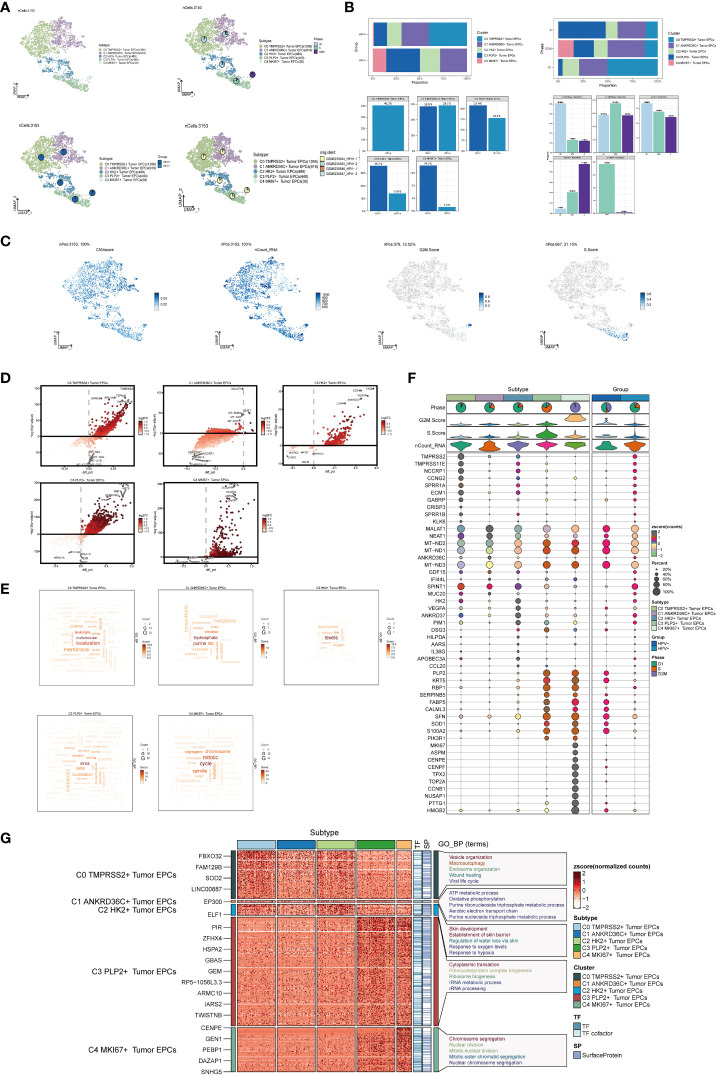
Visualization of cervical cancer tumor epithelial cell subpopulations. **(A)** UMAP diagram demonstrated the 5 cell subpopulations of tumor epithelial cells in cervical cancer patients and the number of cells in each cell subpopulation (upper left); UMAP diagram demonstrated the percentage of different cell cycles in the 5 cell subpopulations (upper right); UMAP diagram demonstrated the distribution of the HPV+ group and the HPV- group in the 5 cell subpopulations (lower left); and UMAP diagram demonstrated the patient origin of the 5 cell subpopulations (lower right). Each point corresponded to a single cell colored according to cell subpopulation. **(B)** Bar graph showed the percentage of each cell subpopulation in HPV+ and HPV- groups (upper left); bar graph showed the percentage of each cell subpopulation at different tumor stages (upper right); bar graph showed, in each cell subpopulation, the percentage of HPV+ cells versus HPV- cells (lower left); and bar graph showed, in each cell subpopulation, the percentage of cells with different cell cycles (lower right). **(C)** UMAP plots visualized the relevant features of 5 cell subpopulations (C0:TMPRSS2+ Tumor EPCs, C1: ANKRD36C+ Tumor EPCs, C2: HK2+ Tumor EPCs, C3: PLP2+ Tumor EPCs, C4:MKI67+ Tumor EPCs): CNVscore, nCount_RNA, S.score, G2M.score. **(D)** Volcano plots demonstrated the expression of differential genes in five cellular subpopulations. **(E)** Word cloud diagrams showed gene pathway enrichment in 5 cell subpopulations. The size of the letters indicated the number of enriched pathways, and the color indicated the high or low score of enriched pathways in different cell subpopulations. **(F)** Bubble graph showed differential expression of Top10 maker genes in 5 cell subpopulations of tumor epithelial cells. The color of the bubbles was based on the normalized data and the size indicated the percentage of genes expressed in the subpopulation. **(G)** GO-BP enrichment analysis demonstrated the biological processes associated with the 5 cell subpopulations.

Next, we used inferCNV (**Supplementary Figure 1**) to explore the single-cell RNA-seq data from tumor, distinguished tumor epithelial cells, and made further sub-clustering, resulting in five cell subgroups and marking their cell numbers: C0 TMPRSS2+ Tumor EPCs(1266), C1 ANKRD36C+ Tumor EPCs(919), C2 HK2+ Tumor EPCs(489), C3 PLP2+ Tumor EPCs(440), C4 MKI67+ Tumor EPCs(39) (**Figure 2A**, upper left), and showed the relationship between five cell subgroups and cell cycle stages (**Figure 2A**, upper right), HPV infection (**Figure 2A**, lower left) and sample source (**Figure 2A**, lower right). The cell subsets of the HPV+ group account for a higher proportion of all cell subsets, which proved that the occurrence of cervical cancer may be related to HPV infection. As previously reported, HPV infection was the main cause of cervical cancer development, which could be seen in 95% of cases (46, 47).The proportion of five cell subgroups in HPV+ group and HPV- group, the proportion of HPV+ and HPV- in five cell subgroups, the proportion of five cell subgroups in cell cycle stages and the proportion of cells in five cell subgroups in cell cycle stages were displayed (**Figure 2B**). It was found that C0 existed only in HPV+ group and accounted for the most in G1 phase, while C3 was mostly in HPV- group, and the proportion of cells in S phase was much higher than that in other two phases. Several related features(CNV score, ncount _ RNA, S. score and G2M. score) of five cell subgroups were visualized (**Figure 2C**).The differential genes of five cell subgroups were displayed by volcano diagrams (**Figure 2D**), the enrichment pathways were displayed by word cloud diagrams (**Figure 2E**), and the marker genes (top10) in each cell subgroup were displayed by bubble diagram (**Figure 2F**). The results of enrichment analysis of GO-BP, the differential genes of five cell subgroups, were displayed by thermogram (**Figure 2G**). C0 TMPRSS2+ Tumor EPCs were related to biological processes such as Vesicle organization, Macroautophagy, Endosome organization,etc. C1 ANKRD36C+ Tumor EPCs are related to biological processes such as ATP metabolic process, Oxidative phosphorylation, Purine ribonucleoside triphosphate metabolic process,etc. C2 HK2+ Tumor EPCs are related to biological processes such as Skin development, Establishment of skin barrier, Regulation of water loss via skin, etc. C3 PLP2+ Tumor EPCs are related to biological processes, such as Cytoplasmic translation, Ribonucleoprotein complex biogenesis, Ribosome biogenesis, etc.C4 MKI67+ Tumor EPCs is related to biological processes such as Chromosome segregation, Nuclear division, Mitotic nuclear division, etc.

### Visualization of pseudo-sequential analysis of tumor epithelial cells by CytoTRACE and monocle

**Figure 3:**
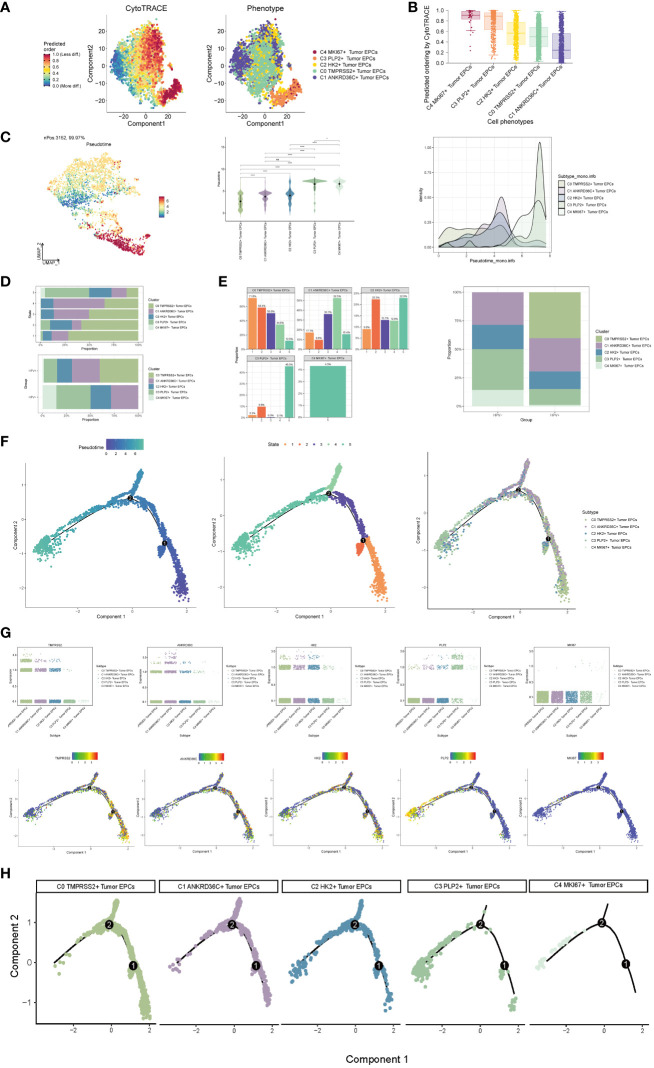
Visualization of pseudotime-series analysis of tumor epithelial cells by CytoTRACE and monocle. **(A)** The left figure represented the analysis of the differentiation of tumor epithelial cells by using CytoTRACE and it was shown in 2D. The color could represent the level of differentiation. The right figure represented the CytoTRACE results displayed according to different tumor epithelial cell subpopulations. The colors represented different tumor epithelial cell subpopulations. **(B)** Box line plot demonstrated the predicted ordering by CytoTRACE of tumor epithelial cell subpopulations. **(C)** UMAP plot, violin plot and ridge plot showed the pseudotime distribution of tumor epithelial cell subpopulations. *, p ≤ 0.05, ****, p < 0.0001 indicated a significant difference, ns indicated a non-significant difference. **(D)** The occupancy of the relevant features of the five tumor epithelial cell subpopulations at different pseudotime stages was visualized: the occupancy of the five tumor epithelial cell subpopulations in different states (state1-state5) (top) and the occupancy of the five cell subpopulations in the HPV+ group and HPV- group (bottom). **(E)** Bar graph showed the occupancy of different states (state1-state5) in 5 tumor epithelial cell subpopulations (left) and the occupancy of 5 cell subpopulations in HPV+ group and HPV- group (right). **(F)** The derivation process of tumor epithelial cells. Left : The figure showed the pseudotime trajectory of tumor epithelial cells; middle: the pseudotime trajectory graph showed the distribution of STATE; right: the pseudotime trajectory graph showed the distribution of tumor epithelial cell subpopulations. **(G)** Scatter plots showed the changes of named genes of 5 cell subpopulations of tumor epithelial cells with the pseudotime sequence (top); pseudotime trajectory plots showed the distribution of named genes of 5 cell subpopulations of tumor epithelial cells on the pseudotime trajectory (bottom). **(H)** Split-plane diagrams of tumor epithelial cell pseudotime sequence trajectories showed the distribution of different cell subpopulations on the pseudotime sequence, respectively.

In order to explore the differentiation and development relationship among the five cell subgroups of tumor epithelial cells, the differentiation of tumor epithelial cells was analyzed and visualized by CytoTRACE (**Figure 3A**). It could be seen that the five cell subgroups differentiated along the direction of C4-C3-C2-C0-C1 (**Figure 3B**).Pseudo-sequential analysis of cancer development process was carried out to explore the differentiation process of tumor epithelial cells. The distribution of epithelial cells from four patients was shown in the pseudotime-series trajectory, and the distribution of cell subgroups in pseudotime-series was shown by using UMAP diagram, violin diagram and ridge diagram respectively. It could be seen that five cell subgroups were continuously differentiated in pseudotime-series (**Figure 3C**). At the same time, five kinds of states were identified. In state1, state2 and state3, the proportion of C0 TMPRSS2+ Tumor EPCs subgroup was the highest, while in state4, the proportion of C1 ANKRD36C+ Tumor EPCs subgroup was the highest, and in state5, the proportion of C3 PLP2+ Tumor EPCs subgroup was the highest, and C4 MKI67+ Tumor EPCs subgroup only existed in state5. In HPV+ and HPV- groups, the proportion of C0 TMPRSS2+ Tumor EPCs subgroup in HPV+ group was the highest, and C3 PLP2+ Tumor EPCs subgroup in HPV- group was the highest (**Figures 3D, E**). In order to study the origin of tumor epithelial cells, the pseudotime sequence trajectory of five cell subgroups was further analyzed. Starting from state1 at the lower right of the trajectory, two trajectories are divided upward, one is state2 downward, the other is continuously divided upward to the second branch point of state3, and the second branch point is divided into two branches, one is state4 upward, and the other is state5 downward to the left. The C0 TMPRSS2+ Tumor EPCs subgroup was mainly displayed at the beginning of the trajectory and in the branch with the first branch point down (corresponding to state1 and state2), the C1 ANKRD36C+ Tumor EPCs subgroup was mainly in the branch with the second branch point up (corresponding to state4), and the C3 PLP2+ Tumor EPCs subgroup mainly existed at the end of the whole pseudotime sequence trajectory (corresponding to state5). Pseudotime series analysis showed that C0 TMPRSS2+ Tumor EPCs subgroup may be the starting point of tumor cells, and gradually differentiated into other subgroups during the progression of cervical cancer (**Figure 3F**).

The named genes of five cell subgroups were selected and their changes with pseudotime series were shown by scatter plots and pseudotime series UMAP plots respectively. It could be seen that the C0 subgroup represented by the gene TMPRSS2 was mostly in the initial state of pseudotime series. The C1 subgroup represented by ANKRD36C and the C2 subgroup represented by HK2 basically run through the pseudotime series. However, the C3 subgroup represented by gene PLP2 and C4 subgroup represented by gene MKI67 were mostly at the end of pseudotime series. The sectional views of each subgroup also confirmed this conclusion (**Figures 3G–H**).

### Slingshot analysis of pseudotime sequence trajectory of tumor epithelial cell subgroups

**Figure 4:**
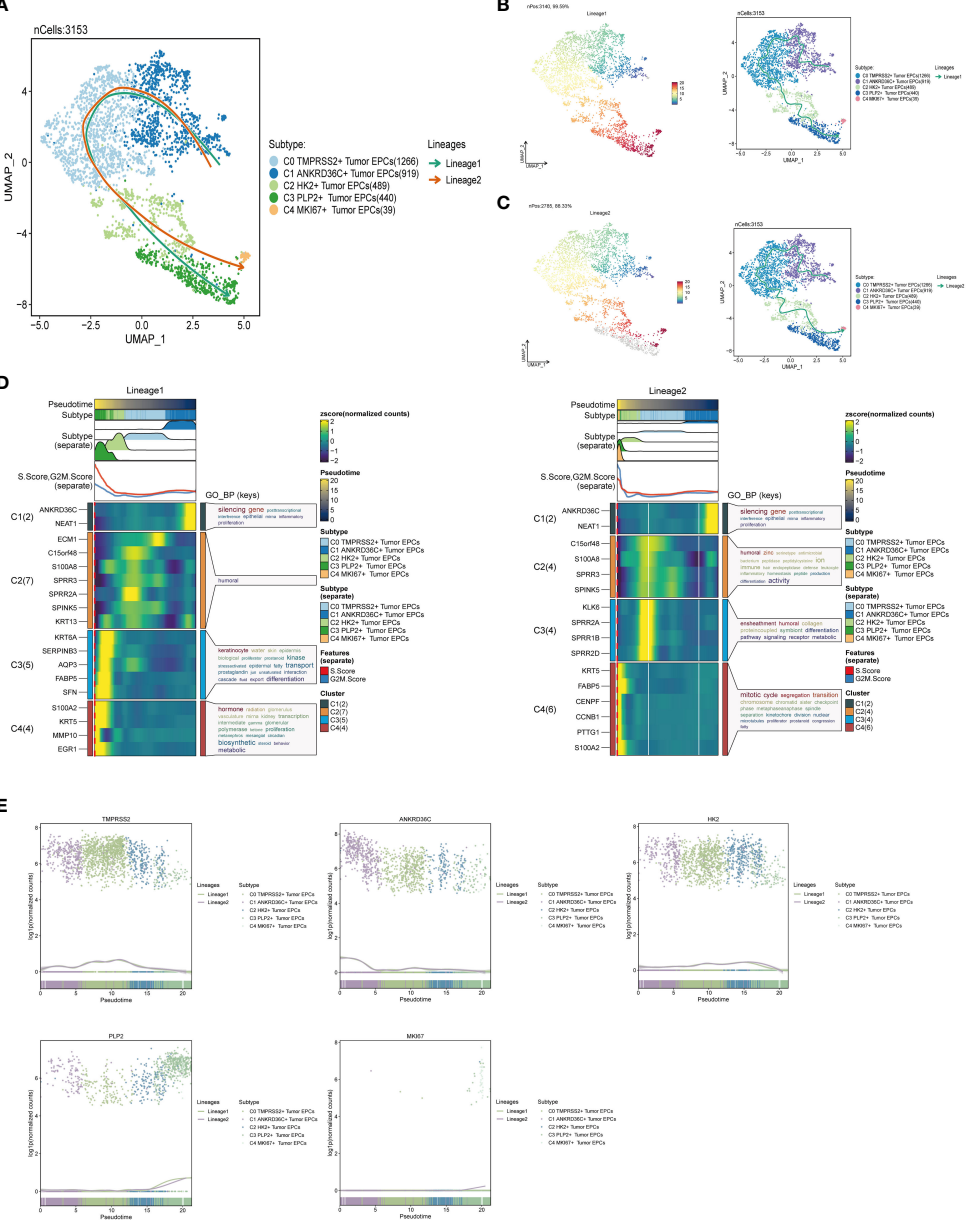
Slingshot analysis of tumor epithelial cell subpopulations on pseudotime trajectories. **(A)** UMAP plot showed the distribution of two differentiation trajectories of tumor epithelial cells fitted by the pseudotime order in all tumor epithelial cells. **(B)** UMAP plot demonstrated the change of Lineage1 with the fitted pseudotime order (left); UMAP plot demonstrated the differentiation trajectory of Lineage1 on the fitted pseudotime order (right). **(C)** UMAP plot demonstrated the change of Lineage2 with the fitted pseudotime order (left); UMAP plot demonstrated the differentiation trajectory of Lineage2 on the fitted pseudotime order (right). **(D)** GO-BP enrichment analysis demonstrated the biological processes corresponding to the two pseudotime trajectories of tumor epithelial cell subpopulations. Left: Lineage1; Right: Lineage2. **(E)** Scatter plots demonstrated the trajectories of named genes of five cell subpopulations of tumor epithelial cells changing on two lineages obtained after slingshot visualization.

Slingshot is a uniquely robust and flexible tool which combines the highly stable techniques necessary for noisy single-cell data with the ability to identify multiple trajectories. In order to infer the continuous branching pedigree structure in tumor epithelial cell data, the pseudotime series trajectories of five cell subgroups were analyzed by using slingshot, and two lineages were obtained: lineage1 and lineage2. The two lineages have similar trajectories, but the final footholds are different, lineage1 finally reaches the C3 PLP2+ Tumor EPCs, and lineage2 finally reaches the C4 MKI67+ Tumor EPCs (**Figure 4A**). Next, the relationship between two lineages and pseudotime-series differentiation trajectory was displayed respectively, and the two pseudotime-series trajectories were visualized by GO-BP enrichment analysis. It was found that C1 in lineage1 was related to biological processes such as silencing gene, C2 was related to biological processes such as humoral, C3 was related to biological processes such as keratinocyte and transport, and C4 was related to biological processes such as hormone and biosynthetic. In lineage2, C1 was related to biological processes such as silencing gene, C2 was related to biological processes such as humoral and activity, C3 was related to biological processes such as ensheathment, humoral, collagen and proteincoupled, and C4 was related to biological processes such as mitotic and cycle (**Figures 4B–D**). Finally, the distribution status of different subpopulations on lineage1 and lineage2 and the differentiation curves with the pseudotime series were shown in scatter plots (**Figure 4E**).

### CellChat analysis between cells

**Figure 5:**
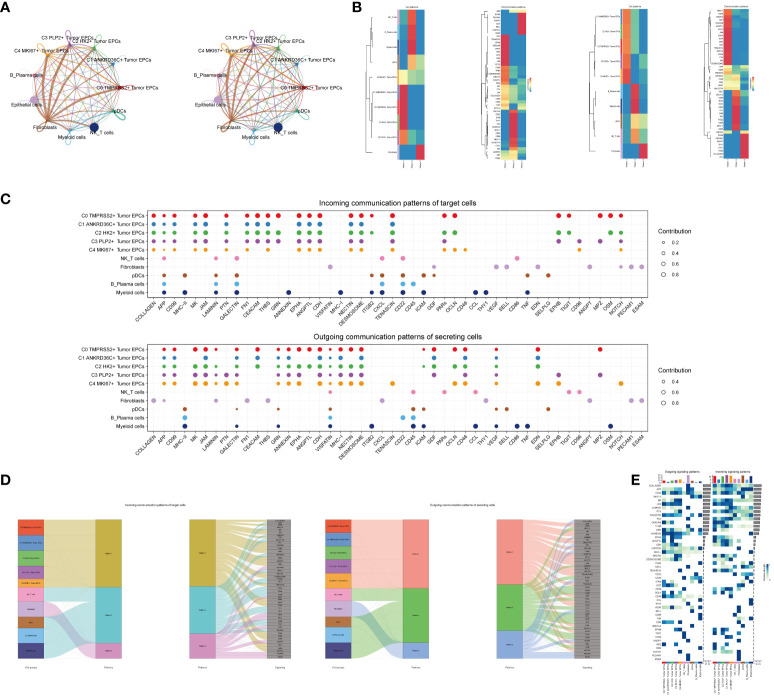
CellChat analysis among all cells. **(A)** Circle plots showed the number (left) and strength (right) of interactions between all cells. **(B)** Heatmap showed pattern recognition of incoming cells (left), and outgoing cells (right) among all cells. **(C)** Outgoing contribution bubble plot and incoming contribution bubble plot showed cellular communication patterns among various cell subpopulations of tumor epithelial cells and other cells. **(D)** Sankey diagrams showed inferred outgoing communication patterns of secreting cells, showed correspondence between inferred potential patterns, cell populations, and signaling pathways. Left: incoming Sankey diagram, right: outgoing Sankey diagram. **(E)** Heatmap showed incoming and outgoing signaling intensities for the all cellular interactions.

In order to systematically elucidate the complex cellular responses, we attempted to probe the cell-to-cell relationships and ligand-receptor communication networks to better understand the interactions between cells. Through CellChat analysis, first, we established intercellular communication networks between most cells, including Myeloid cells, Fibroblasts, NK_T cells and various subgroups of tumor epithelial cells, etc. Then calculated the number of interactions (indicated by the “line” connection between two cell types, the thicker the line, the higher the number of interaction pathways) and the strength of interactions (indicated by the “line” weight, the thicker the line, the stronger the interaction strength) (**Figure 5A**).

We used gene expression pattern analysis methods available on CellChat to explore how cells and signaling pathways interact. First, we determined the correspondence between inferred potential communication patterns and groups of secreted cells to decipher the outgoing communication patterns. Three signaling patterns were identified: pattern 1 (tumor epithelial cells), pattern 2 (NK/T cells, B_Plasma cells, Myeloid cells, pDCs) and pattern 3 (Fibroblasts) (**Figure 5B**). Then, to identify the key incoming and outgoing signals associated with the five tumor epithelial cell subpopulations, the ligand receptor network was quantitatively measured using CellChat to predict its key incoming and outgoing signals by utilizing pattern recognition methods. For example, in cervical cancer, each cell type could be a secreting cell (signal sender) that releases different cytokines or ligands, and each cell type could also be a targeting cell (signal receiver), and ligand-receptor-mediated communication between different cell types should contribute to cervical cancer when receptors on these cells were targeted by ligands released from the same type of cell or from other cells development (**Figure 5C**). In addition to exploring the detailed communication of individual pathways, an important question was how multiple cell populations and signaling pathways coordinate their functions. To address this question, CellChat used a pattern recognition method based on non-negative matrix decomposition to identify global communication patterns, as well as key signals in different cell groups. The application of this analysis revealed three incoming signaling patterns and three outgoing signaling patterns. For example, this output showed that the majority of outgoing tumor epithelial cell signaling was characterized by mode 1, which represented multiple pathways, including but not limited to APP, CD99, CDH, ANNEXIN, etc. All output NK_T cells, B_Plasma cells, Myeloid cells, pDCs signalings were characterized by mode 2, which represented pathways such as CD22, CD45, ICAM, CESC L and TNF. On the other hand, the communication patterns of the target cells suggested that incoming tumor epithelial cell signalings were dominated by mode 1, which included signaling pathways such as CD99 and MK, as well as PTN, CEACAM, CD96, and GRN. The majority of incoming NK_T cells, B_Plasma cells, Myeloid cells, and pDCs signalings were characterized by mode 2, driven by pathways such as APP and ANNEXIN (**Figure 5D**).We found that CD99 can be secreted by almost all types of cells in cervical cancer, and its main target cells (receptors) were tumor epithelial cell subgroups, Fibroblasts and pDCs, among which C3 PLP2+ Tumor EPCs subgroups were most significantly expressed (**Figure 5E**).

### Analysis of CD99 signal pathway

**Figure 6:**
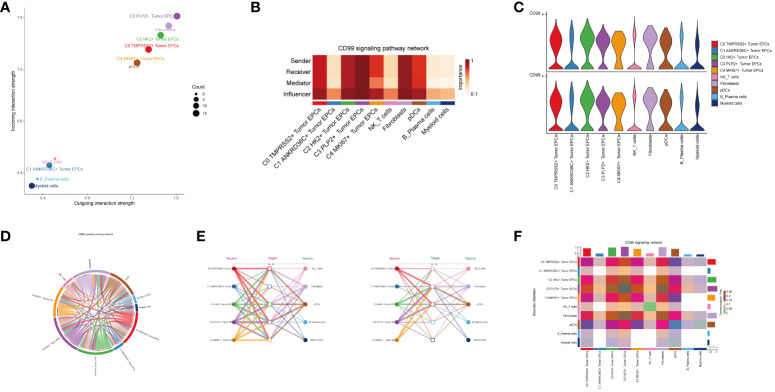
Visual analysis of CD99 signaling pathway. **(A)** Scatter plot showed the cellular communication patterns of CD99 signaling pathway. Each dot represented the communication network of a signaling pathway. The size of the dots indicated the number of signaling pathways. Different colors represented different signaling pathway groups. **(B)** Heatmap demonstrated the centrality score of the CD99 signaling pathway network, showing the relative importance of each cell group. **(C)** Violin plot showed the cellular interactions of the CD99 signaling pathway. **(D)** Circle plot showed the cellular interactions of the CD99 signaling pathway when tumor epithelial cells were selected as the RECEIVER. **(E)** Hierarchical diagram showed the interactions between tumor epithelial cells and other cells in the CD99 signaling pathway. Solid and hollow circles indicated source and target cell types, respectively. The edge color of the middle circle was consistent with the signal source. **(F)** Heatmap showed the cell interactions of the CD99 signaling pathway.

In order to explore the function way of CD99 signal pathway, the CD99 signal pathway was visually analyzed. The cell communication pattern of CD99 signaling pathway was displayed by scatter plot, and it could be seen that the tumor epithelial cell subgroup C3 PLP2+ Tumor EPCs had a large number and the highest intensity on CD99 signaling pathway (**Figure 6A**).

Besides the sender and receiver of CD99 signaling, we also identified the cell types as the medium and influencer of CD99 signaling-mediated intercellular communication according to the relative importance of each cell type based on the algorithm, which was called “centrality measurement”. As could be seen from the figure, the tumor epithelial cell subgroup C3 PLP2+ Tumor EPCs had the highest expression on the CD99 signaling pathway (**Figure 6B**). Violin diagram showed the interaction between cells, and it was found that the epithelial cell subgroup C3 PLP2+ Tumor EPCs was highly expressed on CD99. Combined with the previous experimental results in this paper, it could be concluded that the epithelial cell subgroup C3 PLP2+ Tumor EPCs was an important subgroup of tumor epithelial cells (**Figure 6C**).

The ligand-receptor between tumor epithelial cells and other cells was displayed by chord diagram (**Figure 6D**). If we set all 10 identified cell types in cervical cancer as the source cells of CD99, and set the five cell types listed on the left in **Figure 6E** as potential target cells, Then the hierarchical diagram showed that CD99 released by all 10 cell types could target C0 TMPRSS2+ Tumor EPCs subgroup, C2 HK2+ Tumor EPCs subgroup and C3 PLP2+ Tumor EPCs subgroup, and if the other five cell types listed on the right side of **Figure 6E** were set as potential target cells, Then the layered map showed that CD99 released by all 10 cell types could target C1 ANKRD36C+ Tumor EPCs subgroup and C2 HK2+ Tumor EPCs subgroup (**Figure 6E**). These results indicated that all cell types in cervical cancer may be the source of CD99, but only C0 TMPRS2+ Tumor EPCs subgroup, C1 ANKRD36C+ Tumor EPCs subgroup, C2 HK2+ Tumor EPCs subgroup and C3 PLP2+ Tumor EPCs subgroup had different targeting intensities (line width between cells) for CD99 (**Figure 6E**). The specific situation of cell-to-cell interaction in CD99 signaling pathway was shown in the figure (**Figure 6F**).

### Screening the genes that constitute the risk score and performing correlation analysis

**Figure 7:**
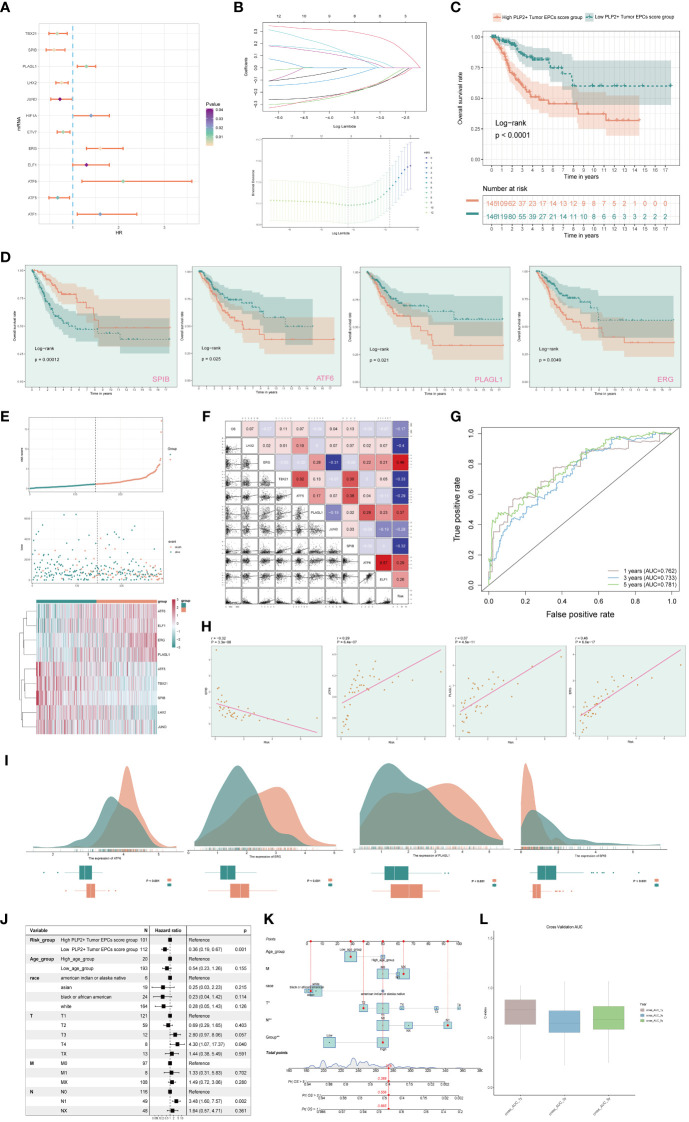
Screening genes which constituted PLP2+ Tumor EPCs score, and grouped into two groups of high and low PLP2+ Tumor EPCs score group and took correlation analysis. **(A)** Forest plot showed univariate COX analysis of genes constituting PLP2+ Tumor EPCs score. HR < 1,protective factor, HR > 1,risk factor. **(B)** LASSO regression profiled nine genes in the TCGA cohort: ERG, ELF1, ATF6, PLAGL1, ATF5, TBX21, SPIB, LHX2, and JUND (top); coefficient profiled were generated based on the logarithmic (lambda) sequence. Selected the optimal parameters (lambda) in the LASSO model (bottom). **(C)** Based on the median PLP2+ Tumor EPCs score, all datas were divided into two groups: high and low PLP2+ Tumor EPCs score group, and Kaplan Meier curve showed the survival analysis of the two groups. **(D)** Kaplan Meier curves showed the overall survival (OS) of cervical cancer (CC) patients with high and low expression of four statistically significant genes (SPIB, ATF6, PLAGL1, and ERG) in all genes which constructed the PLP2+ Tumor EPCs score (p<0.05). **(E)** Curve plots showed hazard scores of high and low PLP2+ Tumor EPCs score groups (top, middle); heatmap showed differential gene expression of high and low PLP2+ Tumor EPCs score group. The color scale was based on normalized data (bottom). Green indicated low PLP2+ Tumor EPCs score group and orange indicated high PLP2+ Tumor EPCs score group. **(F)** Heatmap and scatterplot showed the correlation analysis of genes constituting PLP2+ Tumor EPCs score. Red indicated positive correlation, blue indicated negative correlation, and color shades indicated high or low correlation. **(G)** The ROC curve of the survival plot. The area under the curve (AUC) was 0.762, 0.733 and 0.781 for 1, 3 and 5 years, respectively. **(H)** Scatter plots showed the correlation analysis of modeled genes (4 statistically significant genes) with PLP2+ Tumor EPCs score. **(I)** Peak and box plot showed the different expression among the 4 statistically significant genes which constituted PLP2+ Tumor EPCs score in high and low PLP2+ Tumor EPCs score groups. **(J)** Forest plot showed the multivariate Cox analysis of genes constituting PLP2+ Tumor EPCs score. HR < 1,protective factor, HR > 1, risk factor. **(K)** Column line plot was constructed according to TCGA patient race, T-stage, N-stage, M-stage and etc. **(L)** Box-and-line plot for internal cross-validation of AUC scores at 1, 3, and 5 years.

In order to study the clinical effect of the cell types identified in this study, we analyzed the marker gene (top100) of C3 tumor epithelial cell subgroup by univariate COX analysis. The results showed that there were 12 genes related to the prognosis of patients, among which PLAGL1, HIF1A, ERG, ELF1, ATF6 and ATF1 were risk factors, while TBX21, SPIB, LHX2, JUND, ETV7 and ATF5 were protective factors. In order to avoid multicollinearity of these genes, lasso regression was used for further screening, and nine genes constituting PLP2+ Tumor EPCs score were selected. Lambda diagram verified the above conclusion (**Figure 7B**). Next, the nine PLP2+ Tumor EPCs score genes were divided into high PLP2+ Tumor EPCs score group and low PLP2+ Tumor EPCs score group for survival analysis (**Figure 7C**). Compared with low PLP2+ Tumor EPCs score group, the prognosis of high PLP2+ Tumor EPCs score group was worse. As we expected, high PLP2+ Tumor EPCs score was associated with worse clinical outcome, while low PLP2+ Tumor EPCs score was associated with better clinical outcome (P < 0.0001).

In addition, we also analyzed the survival of genes which constituted PLP2+ Tumor EPCs score (**Figure 7D**), and the results showed that only four of them (SPIB, ATF6, PLAGL1, ERG) were statistically significant (P<0.05). Among them, in the survival analysis chart corresponding to three genes, ATF6, PLAGL1 and ERG, all the groups with high gene expression were associated with worse clinical results. The group with low gene expression was associated with better clinical outcome, while the result of SPIB corresponding to survival analysis chart was just the opposite, which could prove the result in **Figure 7A**, that was, ATF6, PLAGL1 and ERG were risk factors and SPIB was protective factors.

Through the above analysis, two groups of high PLP2+ Tumor EPCs score group and low PLP2+ Tumor EPCs score group had been obtained, and then these two groups were analyzed. The PLP2+ Tumor EPCs score of each patient in TCGA-CESC data set was calculated according to the expression level and regression coefficient of nine genes which established the model. The distribution of PLP2+ Tumor EPCs score in TCGA-CESC dataset was shown in the figure. According to the median PLP2+ Tumor EPCs score, patients in TCGA-CESC cohort were divided into high PLP2+ Tumor EPCs score group and low PLP2+ Tumor EPCs score group. In addition, the distribution of survival time showed that the higher PLP2+ Tumor EPCs score, the worse the prognosis. The corresponding expression levels of nine modeling-related genes were also shown. high PLP2+ Tumor EPCs score group highly expressed ATF6, ELF1, ERG and PLAGL1 genes, while low PLP2+ Tumor EPCs score group highly expressed ATF5, TBX21, SPIB, LHX2 and JUND genes (**Figure 7E**). The correlation among survival days, risk score and genes constituting the model was studied. OS was negatively correlated with PLP2+ Tumor EPCs score, ERG was significantly negatively correlated with JUND, and most other modeling genes were positively correlated. The scatter plot further showed the correlation between nine modeling genes and risk score and OS (**Figure 7F**).

According to the predicted AUC scores of survival ROC curve in 1 year, 3 years and 5 years, the AUC(1 year): 0.762, AUC (3 years): 0.733 and AUC(5 years): 0.781 were obtained (**Figure 7G**). The relationship between four statistically significant modeling genes and PLP2+Tumor EPCs Score was shown by scatter plot (**Figure 7H**), and the difference of expression levels of four statistically significant modeling genes between high PLP2+ Tumor EPCs score group and low PLP2+ Tumor EPCs score group was shown (**Figure 7I**). It was found that ATT6, ERG and PLAGL1 were more expressed in high PLP2+ Tumor EPCs score group, while SPIB was more expressed in low PLP2+ Tumor EPCs score group (P < 0.001). The differential expression of genes in high PLP2+ Tumor EPCs score group and low PLP2+ Tumor EPCs score group, and the differential expression in different ages, different T, N, M stages and different races were demonstrated by box plots (**Supplementary Figure 2**).

In order to verify the independence of risk factors, a gene-cell clinical prediction model was constructed, and multi-factor Cox regression was carried out by combining age, race, T stage, N stage and M stage clinicopathological factors with high PLP2+ Tumor EPCs score group and low PLP2+ Tumor EPCs score group, with a p value of 0.001, indicating that PLP2+Tumor EPCs Score could be independent risk factor (**Figure 7J**). Age, race, T, N and M stages were selected to construct the nomogram, which showed the 1-year survival rate, 3-year survival rate and 5-year survival rate (**Figure 7K**). The estimated survival rates of one of these patients in 1, 3 and 5 years are 0.865, 0.556 and 0.389, respectively. In order to further evaluate the accuracy of the nomogram, the box diagram was used to show the internal cross-validation results (**Figure 7L**).

### Analysis of the difference of immune infiltration between high PLP2+ Tumor EPCs score group and low PLP2+ Tumor EPCs score group.

**Figure 8:**
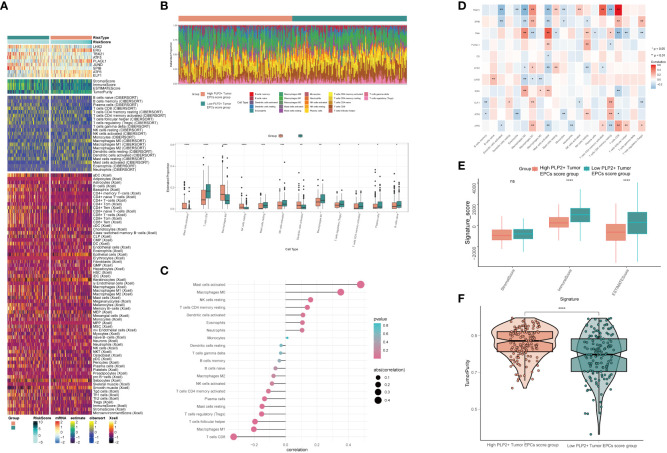
Differential analysis of immune infiltration in high and low PLP2+ Tumor EPCs score group **(A)** Heatmap showed differential expression of immune infiltration in high and low PLP2+ Tumor EPCs score group. **(B)** Stacked bar graph of immune infiltration (top); box-and-line plot showed the differential immune infiltration of 12 immune cells which had significant differences in high and low PLP2+ Tumor EPCs score groups (bottom). **(C, D)** Bar graph and heatmap showed the correlation analysis of immune cells with genes constituting PLP2+ Tumor EPCs score. **(E)** Box line plot showed the difference between high and low PLP2+ Tumor EPCs score groupin StromalScore, ImmuneScore, and ESTIMATEScore. **(F)** Violin plot showed the differences of TumorPurity in high and low PLP2+ Tumor EPCs score group. *, p < 0.05; **, p < 0.01; ***, p < 0.001; ****, p < 0.0001 indicated a significant difference and ns indicated a non-significant difference.

In order to explore the immune infiltration in high PLP2+ Tumor EPCs score group and low PLP2+ Tumor EPCs score group, and to observe the relationship between immune infiltrating cells and the two groups, we showed the differential expression of immune infiltration between the two groups by thermogram (**Figure 8A**). In order to explore the immune infiltration of high PLP2+ Tumor EPCs score group and low PLP2+ Tumor EPCs score group, the predicted abundance of different immune cells was displayed (upper **Figure 8B**). We used CIBERSORT algorithm to determine the immune cell infiltration of cervical cancer patients from TCGA database, and show the predicted abundance of 12 immune cells with differences between the two groups. High PLP2+ Tumor EPCs score group was more common in Mast cells activated, Macrophages M0, Dendritic cells activated, etc., while low PLP2+ Tumor EPCs score group was more common in T cells CD8, T cells regulatory, B cells naive, etc. (under **Figure 8B**). The correlation between immune infiltrating cells and PLP 2+Tumor EPCs score was demonstrated by bar chart. PLP2+ Tumor EPCs score was positively correlated with Mast cells activated, Macrophages M0, Dendritic cells activated, and negatively correlated with T cells CD8, T cells regulatory, B cells naive, etc. (**Figure 8C**). Through a variety of methods for evaluating the content of immune cells, the relationship between the nine genes, OS and PLP2+ Tumor EPCs score and immune cells was compared and summarized, and displayed by thermal diagram. The closer the color was to red, the higher the positive correlation, and the closer the color was to blue, the higher the negative correlation (**Figure 8D**).

Stromal Score, Immune Score and EATIMATE Score of high PLP2+ Tumor EPCs score group and low PLP2+ Tumor EPCs score group were displayed, and it could be obtained that the results of Stromal Score Group were not statistically significant. Immune Score and EATIMATE Score were both low PLP2+ Tumor EPCs score group higher than high PLP2+ Tumor EPCs score group (**Figure 8E**). Visualizing the Tumor Purity of two groups, the value of Tumor Purity of high PLP2+ Tumor EPCs score group was higher (**Figure 8F**).

### Difference analysis and enrichment analysis

**Figure 9:**
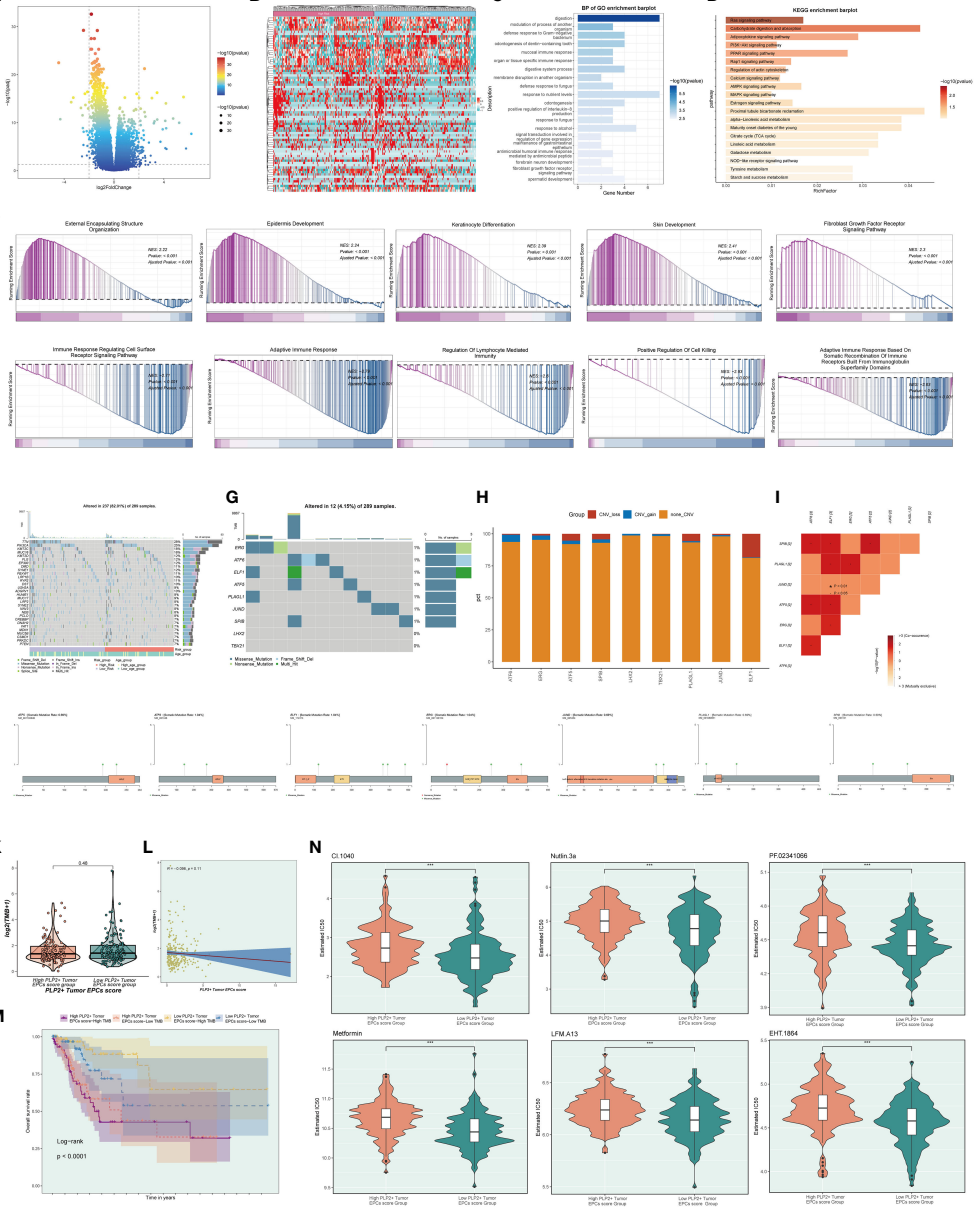
Gene mutations of tumor epithelial cell.s **(A, B)** Volcano plot and heatmap showed the expression of differential genes in the high and low PLP2+ Tumor EPCs score group. **(C)** Bar graph showed the results of all GO-BP enrichment analysis. **(D)** Results of enrichment on different pathways were shown by KEGG enrichment analysis of differential genes. **(E)** Enrichment score values on different pathways were displayed by GSEA scoring of GO-BP enrichment entries for differential genes. **(F)** Mutation waterfall plot showed the differences in the top 30 most frequently mutated genes in the somatic cells between the two groups. The upper bars indicated the mutation load for each sample and the right bars indicated the total percentage of mutations of the genes in these samples. **(G)** Mutation waterfall plot showed mutations load of the genes that constituted the PLP2+ Tumor EPCs score in the samples. The upper bars indicated the mutation load for each sample, and the right bars indicated the total proportion of mutations of this gene in these samples. **(H)** Bar graph showed the results of predicting chromosome gains and losses in TCGA samples. Blue color indicated chromosome copy number gain; red color indicated chromosome copy number loss; orange color indicated no change in chromosome copy number. **(I)** Heatmap showed the correlation of mutation profiles of genes that constituted the PLP2+ Tumor EPCs score. **(J)** Lollipop chart visualized the mutation analysis of genes. **(K)** Box-and-line plot showed the differences of mutation load in high and low PLP2+ Tumor EPCs score groups. **(L)** Scatter plot showed the correlation analysis between mutation load and PLP2+ Tumor EPCs score. **(M)** Scoring according to tumor mutation load,all datas were divided into four groups: high-risk high mutation load, high-risk low mutation load, low-risk high mutation load, and low-risk low mutation load, and the curve showed the survival analysis results of the four groups. **(N)** Violin plots showed the differences of different drug sensitivities in the high and low PLP2+ Tumor EPCs score group. ***, p < 0.001 indicated a significant difference.

In order to explore the differences between high PLP2+ Tumor EPCs score group and low PLP2+ Tumor EPCs score group, the following analysis was made. First, the volcano diagram and thermal diagram were used to show the expression of differential genes between the two groups (**Figures 9A, B**). In order to further determine the potential role of each subgroup in the initiation and progression of cervical cancer, functional enrichment was carried out, and the GO-BP enrichment analysis results of differential genes were displayed with bar charts. The results showed that the differential genes were mainly related to digestion, modulation of process of another organism, defense response to gram negative bacteria and odontogenesis of dentition containing tooth (**Figure 9C**). KEGG enrichment analysis was carried out on the differential genes, and the enrichment results of different pathways were displayed by bar graphs, and it was found that the differential genes were enriched with Ras signaling pathway, carbohydrate digestion and absorption, PPAR signaling pathway and other pathways (**Figure 9D**). Through GSEA scoring of GO-BP enrichment items of different genes, the enrichment scores of genes in different pathways were displayed (**Figure 9E**).

### Mutation analysis

To determine the correlation between gene mutations and immune components in TME, we initiated further studies, first showing the top 30 most frequently mutated genes in two groups of somatic cells. The upper bars indicated the mutation load for each sample, and the right bars indicated the total percentage of mutations in that gene in those samples (**Figure 9G**). The cellular mutation data from the two groups were analyzed and visualized to show the mutations in the nine genes that were modeled (**Figure 9F**), and the chromosomal gains and losses were demonstrated by using bar graphs to show that the most CNV gain was seen in ATF6, whereas the most CNV loss was seen in ELF1 (**Figure 9H**). Heatmaps were used to show the correlation of mutation profiles among the genes constituting the risk score (**Figure 9I**).

And lollipop plots were used to visualize the mutation profiles of different genes (**Figure 9J**). To explore the difference situation of mutation load between high PLP2+ Tumor EPCs score group and low PLP2+ Tumor EPCs score group, the results were visualized and analyzed by using violin plots, which were not statistically significant (**Figure 9K**), and the correlation between mutation load and risk scores was demonstrated by using scatter plots, which were not statistically significant (**Figure 9L**). According to the tumor mutation load score, divided into four groups: high-risk high mutation load, high-risk low mutation load, low-risk high mutation load, and low-risk low mutation load, and the curves showed the results of the survival analysis of the four groups: the low-risk high mutation load group had the best survival, while the high-risk high mutation load group had the worst survival (p < 0.0001) (**Figure 9M**).

### Drug sensitivity analysis

Finally, the differences in different drug sensitivities in the high PLP2+ Tumor EPCs score group versus the low PLP2+ Tumor EPCs score group were shown by violin plots (**Figure 9N**). For example, CI.1040, Nutlin.3a, PF.02341066, Metformin, EHT.1864, and LFM.A13 had higher drug sensitivities in high PLP2+ Tumor EPCs group than in low PLP2+ Tumor EPCs score group.

### Experimental verification

**Figure 10:**
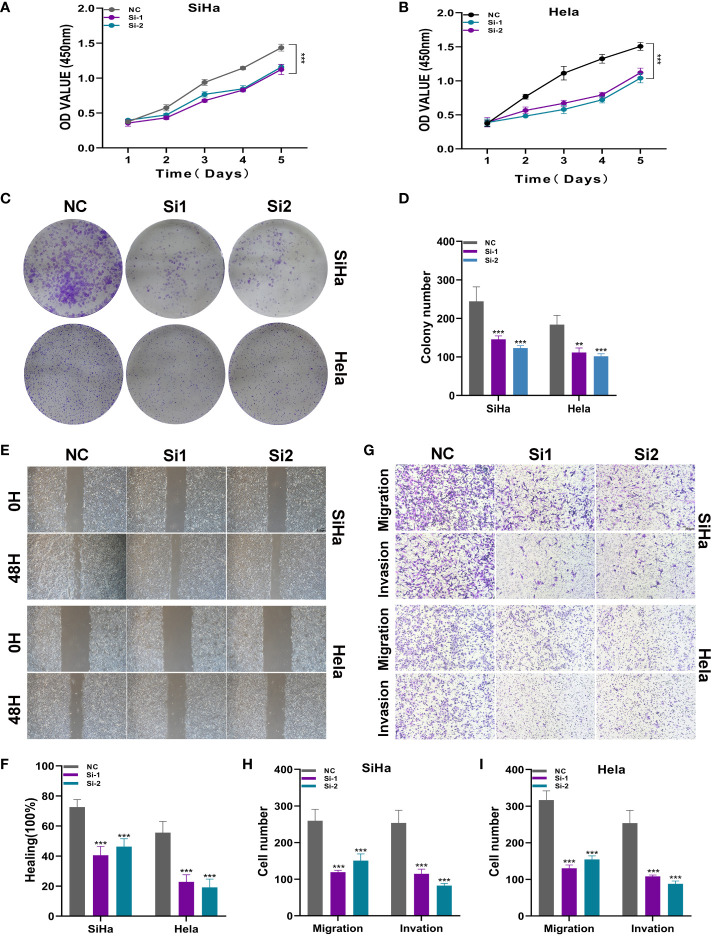
ATF6 significantly affects the proliferation and migration of cervical cancer cell lines. **(A, B)** CCK-8 experiment. After ATF6 knockdown, the proliferation ability of SiHa and Hela cell lines decreased significantly. **(C, D)** Plate cloning experiment. After ATF6 knockdown, the colony formation ability of SiHa and Hela cell lines decreased significantly. **(E, F)** wound healing test. After ATF6 knockdown, the migration ability of SiHa and Hela cell lines decreased significantly. **(G–I)** Transwell experiment. After ATF6 knockdown, the migration and invasion ability of SiHa and Hela cell lines were significantly reduced. (**P<0.01; ***P< 0.001).

To further elucidate the function of ATF6, we conducted *in vitro* functional experiments. As shown in **Figures 10A, B**, the CCK8 experiment showed that compared with the control group, the proliferation capacity of the two cell lines in the ATF6 knockdown group was significantly decreased. The results of plate cloning showed that the number and size of colony formation in both cell lines were significantly inhibited after ATF6 gene knockdown (**Figures 10C, D**). Further, the inhibitory effect of ATF6 knockdown on the proliferation of two cervical cancer cell lines was demonstrated. The subsequent wound healing experiment results showed that the cell migration rate was slower in the ATF6 knockdown group, and the results were statistically significant (**Figures 10E, F**). The results of the Transwell experiment showed that the number of cells penetrating the lower chamber was significantly reduced after ATF6 gene knockdown, indicating that ATF6 gene knockdown significantly inhibited the migration and invasion ability of cervical cancer cells (**Figures 10G–I**).

## Discussion

In recent years, the rapid advancement of bioinformatics has profoundly accelerated the diagnosis and prognosis of diseases (48–50). In this investigation, we employed single-cell RNA sequencing (scRNA-seq) to comprehensively delineate the cellular heterogeneity of human CC. Leveraging scRNA-seq technology, we identified all cellular phenotypes present in CC, including NK_T cells, epithelial cells, fibroblasts, plasmacytoid dendritic cells (pDCs), B plasma cells, and myeloid cells. Among these, epithelial cells emerged as the predominant cellular population. Drawing upon prior research indicating the pivotal role of squamous epithelial cell dysfunction in the initiation of cervical cancer (51), our focus shifted towards the investigation of epithelial cells. Through inferCNV analysis, we characterized tumor epithelial cells and conducted dimensionality reduction clustering, revealing five distinct cellular subgroups: C0 TMPRSS2+ Tumor Epithelial Progenitor Cells, C1 ANKRD36C+ Tumor Epithelial Progenitor Cells, C2 HK2+ Tumor Epithelial Progenitor Cells, C3 PLP2+ Tumor Epithelial Progenitor Cells, and C4 MKI67+ Tumor Epithelial Progenitor Cells.

Through the application of slingshot, monocle, and cytoTRACE, the differentiation trajectory of tumor epithelial cells along a pseudo-temporal sequence was demonstrated. This analysis identified our targeted subgroup for this study: the C3 PLP2+ Tumor EPCs subgroup. Regarding the nomenclature of this subgroup gene, previous research suggests that PLP2 is a transmembrane protein located in the endoplasmic reticulum (52, 53). It is recognized as an oncogenic inducer in various cancers, including melanoma, osteosarcoma, breast cancer, hepatocellular carcinoma, and acute lymphoblastic leukemia (54). PLP2 has been implicated in accelerating UCEC cell proliferation, the epithelial-mesenchymal transition (EMT) process, invasion, and metastasis, thereby promoting tumor progression (55). It is associated with the persistence and metastasis of tumor cells in melanoma and hematologic malignancies (53). Additionally, PLP2 is known to enhance tumor sphere-forming ability and cell proliferation (56, 57). It can be seen that there is an inevitable link between PLP2 and tumor progression. Therefore, we hypothesize that the C3 PLP2+ Tumor EPCs subgroup is intricately linked to the progression of the tumor.

To explore the interactions between the C3 PLP2+ Tumor EPCs subgroup and other cell types, CellChat communication pattern analysis was employed to unveil coordinated responses among different cell types. Distinct cell types can simultaneously activate either common cell type-independent signaling transduction pathways or distinct cell type-specific signaling transduction pathways. This methodology is utilized for inferring, analyzing, and visualizing intercellular communication from given scRNA-seq data (31). Through the application of CellChat to depict the relationships between the tumor epithelial cell subgroup and other cell types, three patterns were identified along with their corresponding signal pathway expressions. Notably, the CD99 signaling pathway corresponds to Tumor EPCs pattern 1 and is secreted by almost all cell types in cervical cancer, signifying its crucial role as a significant signaling pathway. The presentation of the subgroups on the CD99 signaling pathway reveals that the C3 PLP2+ Tumor EPCs subgroup has the highest quantity and centralization score, providing evidence of a robust association between this pathway and the C3 PLP2+ Tumor EPCs subgroup. This confirmation underscores the significance of the C3 PLP2+ Tumor EPCs subgroup as the focal point in this study.

To substantiate the role of the C3 PLP2+ Tumor Epithelial Progenitor Cells subgroup in tumor advancement, we proceeded with the development of a novel prognostic model using LASSO regression analysis and COX risk regression analysis. This model was designed to elucidate the association between this subgroup and prognosis, with a focus on nine selected genes to establish the PLP2+ Tumor EPCs score. Among the nine genes in the model, PLAG1 is acknowledged as an oncogene with significant DNA binding affinity and overlapping functions. Its involvement in promoter swapping and subsequent activation plays a pivotal role in the pathogenesis of pleomorphic adenomas of the salivary gland, lipoblastomas, and hepatoblastomas (58–60). ERG, in concert with co-repressive proteins such as HDAC and EZH2, governs AR transcriptional activity, suppressing epithelial differentiation and fostering tumor progression (61). ELF1 exhibits heightened expression in glioma tissues and exhibits a close correlation with WHO grading and patient Karnofsky Performance Status (KPS) scores, suggesting its potential role as a tumor-promoting factor (62). ATF6 serves as an inducer of genes that augment protein folding and restore protein homeostasis (63), while also promoting inflammation during the course of chronic pancreatitis (64). SPIB is upregulated in various malignant tumors, including colorectal cancer, hepatocellular carcinoma, and gastric cancer (65).

The constructed PLP2+ Tumor EPCs score has been demonstrated as an independent prognostic factor. Based on the median risk score, it has been divided into two distinct prognostic groups: the high PLP2+ Tumor EPCs score group and the low PLP2+ Tumor EPCs score group. Subsequent construction of ROC curves and column charts, along with comprehensive analysis, indicates that a low PLP2+ Tumor EPCs score is associated with a better prognosis, while a high PLP2+ Tumor EPCs score is conversely related to a poorer prognosis. Therefore, the PLP2+ Tumor EPCs score can serve as a theoretical basis for clinical decision-making.

The immune system plays a pivotal role in carcinogenesis (66). It is widely acknowledged that the migratory capacity of tumor cells is closely associated with poor prognosis and recurrence (67). Immune cells form the cellular foundation for immunotherapy, and gaining in-depth insights into immune infiltration is crucial for uncovering potential molecular mechanisms and providing new immunotherapeutic strategies to enhance clinical outcomes (68). Therefore, to further discuss the role of the PLP2+ Tumor EPCs score in the tumor process, we analyzed the immune infiltration in two groups based on the PLP2+ Tumor EPCs score (high PLP2+ Tumor EPCs score group and low PLP2+ Tumor EPCs score group). The high PLP2+ Tumor EPCs score group was found to have a higher prevalence of activated Mast cells, Macrophages M0, and activated Dendritic cells. In contrast, the low PLP2+ Tumor EPCs score group exhibited a higher prevalence of T cells CD8, regulatory T cells, and naive B cells, mostly associated with favorable prognoses in various cancers (69). Visualization of the analysis results through Stromal Score, Immune Score, ESTIMATE Score, and Tumor Purity revealed that the Stromal Score had no statistical significance. A higher estimated score in Immune Score indicated a greater abundance of immune components in the tumor microenvironment (TME), while the ESTIMATE Score, representing the sum of Immune Score and Stromal Score, reflected the comprehensive proportion of these two components in the TME (70). Tumor purity refers to the proportion of tumor cells in tumor tissue (71). Consequently, the conclusion can be drawn that the low PLP2+ Tumor EPCs score group has a higher total of immune and stromal components, with a lower proportion of tumor cells, potentially correlating with better prognostic outcomes in this group.

The presentation includes differential gene expression, enriched pathways, mutation profiles, and variances in drug sensitivity between the two groups. Notably, drugs such as CI.1040, Nutlin.3a, PF.02341066, Metformin, EHT.1864, and LFM.A13 have undergone investigation in various malignancies, encompassing gastric cancer, breast cancer, endometrial cancer, and prostate cancer (72–75). Metformin has emerged as a promising anti-tumor agent, leading to significant advancements in the management of breast cancer and colorectal cancer (72). Nutlin-3a exhibits immune-modulating properties, rendering it a viable option for tumor therapy (76). PF.02341066 serves as a multi-target inhibitor of anaplastic lymphoma kinase (ALK), ROS1, and MET proto-oncogene receptor tyrosine kinase, and is the first agent approved by the U.S. Food and Drug Administration (FDA) and the European Medicines Agency (EMA) for the treatment of advanced ROS1 fusion-positive lung cancer (77, 78). Based on the analysis of drug sensitivity, it is suggested that heightened sensitivity to drugs may correlate with a high PLP2+ Tumor EPCs score. Our findings from drug sensitivity analysis furnish a foundation for the election of targeted therapies for cervical cancer patients and provide novel insights for the development of innovative targeted therapeutic agents.

Furthermore, our cellular experiments have provided additional evidence regarding the functions of key molecules. However, this study still has certain limitations. Firstly, the sample size selected is relatively small, focusing only on a small subset of CC patients. Secondly, we conducted only transcriptomic studies and *in vitro* experiments. Thirdly, in previous studies, it has been found that there is a link between cervical cancer and HPV infection. In cases of cervical squamous lesions, the hrHPV positivity rate was 55.6% (79). The relationship between the C3 PLP2+ Tumor EPCs and HPV infection can be further explored in the following studies. Fourth, the article mentions the cellular immune infiltration in different groups. Studies have proposed that T lymphocytes can improve the tumor microenvironment of cervical cancer, improve treatment efficacy, and improve prognosis (80, 81), and may continue to explore in this direction in the future. In subsequent research, we plan to validate the role of the PLP2+ Tumor EPCs score in CC across a larger sample size. Additionally, we aim to explore the functions of relevant subgroups and key molecules in various omics data, such as metabolomics, proteomics, and ATAC-seq. We will integrate *in vivo* and *in vitro* experiments to provide more comprehensive validation.

## Conclusion

In summary, our investigation elucidated alterations in the tumor microenvironment and highlighted the pivotal role of the C3 PLP2+ Tumor Epithelial Progenitor Cells subpopulation in the onset and progression of cervical carcinoma (CC). Additionally, we identified an independent prognostic factor, the PLP2+ Tumor EPCs score. Our findings unveil prospective therapeutic targets for CC, offering valuable resources and enhanced understanding of the etiology and progression of the disease.

## Data availability statement

The original contributions presented in the study are included in the article/**Supplementary Material**. Further inquiries can be directed to the corresponding authors.

## Ethics statement

The study protocols were performed according to Declaration of Helsinki.

## Author contributions

ZL: Conceptualization, Data curation, Formal analysis, Investigation, Methodology, Project administration, Resources, Software, Supervision, Validation, Visualization, Writing – original draft, Writing – review & editing. XL: Data curation, Investigation, Project administration, Writing – original draft, Writing – review & editing. HS: Formal analysis, Project administration, Resources, Validation, Writing – review & editing. RC: Conceptualization, Methodology, Project administration, Writing – review & editing. LZ: Data curation, Project administration, Validation, Writing – review & editing. CD: Software, Validation, Writing – review & editing. YS: Formal analysis, Investigation, Methodology, Writing – review & editing. WF: Formal analysis, Project administration, Resources, Writing – review & editing. SW: Funding acquisition, Resources, Validation, Supervision, Writing – review & editing. ZY: Resources, Supervision, Writing – review & editing.

## Supplementary material

Supplementary Figure 1 The InferCNV heatmap illustrates copy number variations in Epithelial cells, using NK T cells as reference cells.

Supplementary Figure 2 The box plots depict the differential expression of modeled genes in the high PLP2+ Tumor EPCs score group and low PLP2+ Tumor EPCs score group, as well as variations in expression across different age groups, T, N, M stages, and ethnicities.

## References

[1] Sung H Ferlay J Siegel RL Laversanne M Soerjomataram I Jemal A. Global cancer statistics 2020: GLOBOCAN estimates of incidence and mortality worldwide for 36 cancers in 185 countries. CA Cancer J Clin. (2021) 71:209–49. doi: 10.3322/caac.21660 33538338

[2] Höhn AK Brambs CE Hiller GGR May D Schmoeckel E Horn LC. 2020 WHO classification of female genital tumors. Geburtshilfe Frauenheilkd. (2021) 81:1145–53. doi: 10.1055/a-1545-4279 PMC8494521 34629493

[3] Rodriguez NM. Participatory innovation for human papillomavirus screening to accelerate the elimination of cervical cancer. Lancet Glob Health. (2021) 9:e582–3. doi: 10.1016/S2214-109X(20)30522-2 PMC8145853 33865467

[4] Sankaranarayanan R Basu P Kaur P Bhaskar R Singh GB Denzongpa P. Current status of human papillomavirus vaccination in India's cervical cancer prevention efforts. Lancet Oncol. (2019) 20:e637–44. doi: 10.1016/S1470-2045(19)30531-5 31674322

[5] Zarbá JJ Jaremtchuk AV Gonzalez Jazey P Keropian M Castagnino R Mina C. A phase I-II study of weekly cisplatin and gemcitabine with concurrent radiotherapy in locally advanced cervical carcinoma. Ann Oncol. (2003) 14:1285–90. doi: 10.1093/annonc/mdg345 12881394

[6] Kitagawa R Katsumata N Shibata T Kamura T Kasamatsu T Nakanishi T. Paclitaxel plus carboplatin versus paclitaxel plus cisplatin in metastatic or recurrent cervical cancer: the open-label randomized phase III trial JCOG0505. J Clin Oncol. (2015) 33:2129–35. doi: 10.1200/JCO.2014.58.4391 25732161

[7] Ferrall L Lin KY Roden RBS Hung CF Wu TC. Cervical cancer immunotherapy: facts and hopes. Clin Cancer Res. (2021) 27:4953–73. doi: 10.1158/1078-0432.CCR-20-2833 PMC8448896 33888488

[8] Stryker ZI Rajabi M Davis PJ Mousa SA. Evaluation of angiogenesis assays. Biomedicines. (2019) 7:37. doi: 10.3390/biomedicines7020037 31100863 PMC6631830

[9] Quail DF Joyce JA. Microenvironmental regulation of tumor progression and metastasis. Nat Med. (2013) 19:1423–37. doi: 10.1038/nm.3394 PMC3954707 24202395

[10] Rhodes DR Chinnaiyan AM. Integrative analysis of the cancer transcriptome. Nat Genet. (2005) 37 Suppl:S31–7. doi: 10.1038/ng1570 15920528

[11] Guo X Zhang Y Zheng L Zheng C Song J Zhang Q. Global characterization of T cells in non-small-cell lung cancer by single-cell sequencing [published correction appears in Nat Med. Nat Med. (2018) 24:978–85. doi: 10.1038/s41591-018-0045-3 29942094

[12] Gladka MM Molenaar B de Ruiter H van der Elst S Tsui H Versteeg D. Single-cell sequencing of the healthy and diseased heart reveals cytoskeleton-associated protein 4 as a new modulator of fibroblasts activation. Circulation. (2018) 138:166–80. doi: 10.1161/CIRCULATIONAHA.117.030742 29386203

[13] Ofengeim D Giagtzoglou N Huh D Zou C Yuan J. Single-cell RNA sequencing: unraveling the brain one cell at a time. Trends Mol Med. (2017) 23:563–76. doi: 10.1016/j.molmed.2017.04.006 PMC5531055 28501348

[14] Fattore L Ruggiero CF Liguoro D Mancini R Ciliberto G. Single cell analysis to dissect molecular heterogeneity and disease evolution in metastatic melanoma. Cell Death Dis. (2019) 10:827. doi: 10.1038/s41419-019-2048-5 31672982 PMC6823362

[15] Shih AJ Menzin A Whyte J Lovecchio J Liew A Khalili H. Identification of grade and origin specific cell populations in serous epithelial ovarian cancer by single cell RNA-seq [published correction appears in PLoS One. PloS One. (2018) 13:e0206785. doi: 10.1371/journal.pone.0206785 30383866 PMC6211742

[16] Bian S Hou Y Zhou X Li X Yong J Wang Y. Single-cell multiomics sequencing and analyses of human colorectal cancer. Science. (2018) 362:1060–3. doi: 10.1126/science.aao3791 30498128

[17] Xu J Liao K Yang X Wu C Wu W. Using single-cell sequencing technology to detect circulating tumor cells in solid tumors [published correction appears in Mol Cancer. Mol Cancer. (2021) 20:104. doi: 10.1186/s12943-021-01392-w 34412644 PMC8375060

[18] Li C Guo L Li S Hua K. Single-cell transcriptomics reveals the landscape of intra-tumoral heterogeneity and transcriptional activities of ECs in CC. Mol Ther Nucleic Acids. (2021) 24:682–94. doi: 10.1016/j.omtn.2021.03.017 PMC8099483 33996252

[19] Han L Wei X Liu C Volpe G Zhuang Z Zou X. Cell transcriptomic atlas of the non-human primate Macaca fascicularis. Nature. (2022) 604:723–31. doi: 10.1038/s41586-022-04587-3 35418686

[20] Stuart T Butler A Hoffman P Hafemeister C Papalexi E Mauck WM 3rd. Comprehensive integration of single-cell data. Cell. (2019) 177:1888–1902.e21. doi: 10.1016/j.cell.2019.05.031 31178118 PMC6687398

[21] Li X Guan H Ma C Dai Y Su J Chen X. Combination of bulk RNA sequencing and scRNA sequencing uncover the molecular characteristics of MAPK signaling in kidney renal clear cell carcinoma. Aging (Albany NY). (2024) 16(2):1414–39. doi: 10.18632/aging.205436 PMC10866414 38217548

[22] Lin Z Sui X Jiao W Chen C Zhang X Zhao J. Mechanism investigation and experiment validation of capsaicin on uterine corpus endometrial carcinoma. Front Pharmacol. (2022) 13:953874. doi: 10.3389/fphar.2022.953874 36210802 PMC9532580

[23] Ding Y Zhao Z Cai H Zhou Y Chen H Bai Y. Single-cell sequencing analysis related to sphingolipid metabolism guides immunotherapy and prognosis of skin cutaneous melanoma. Front Immunol. (2023) 14:1304466. doi: 10.3389/fimmu.2023.1304466 38077400 PMC10701528

[24] Ge Q Zhao Z Li X Yang F Zhang M Hao Z. Deciphering the suppressive immune microenvironment of prostate cancer based on CD4+ regulatory T cells: Implications for prognosis and therapy prediction. Clin Transl Med. (2024) 14:e1552. doi: 10.1002/ctm2.1552 38239097 PMC10797244

[25] Zhang J Peng G Chi H Yang J Xie X Song G. CD8 + T-cell marker genes reveal different immune subtypes of oral lichen planus by integrating single-cell RNA-seq and bulk RNA-sequencing. BMC Oral Health. (2023) 23:464. doi: 10.1186/s12903-023-03138-0 37422617 PMC10329325

[26] Ren Q Zhang P Lin H Feng Y Chi H Zhang X. A novel signature predicts prognosis and immunotherapy in lung adenocarcinoma based on cancer-associated fibroblasts. Front Immunol. (2023) 14:1201573. doi: 10.3389/fimmu.2023.1201573 37325647 PMC10264584

[27] Zhang P Pei S Wu L Xia Z Wang Q Huang X. Integrating multiple machine learning methods to construct glutamine metabolism-related signatures in lung adenocarcinoma. Front Endocrinol (Lausanne). (2023) 14:1196372. doi: 10.3389/fendo.2023.1196372 37265698 PMC10229769

[28] Butler A Hoffman P Smibert P Papalexi E Satija R. Integrating single-cell transcriptomic data across different conditions, technologies, and species. Nat Biotechnol. (2018) 36:411–20. doi: 10.1038/nbt.4096 PMC6700744 29608179

[29] Zhao Z Ding Y Tran LJ Chai G Lin L. Innovative breakthroughs facilitated by single-cell multi-omics: manipulating natural killer cell functionality correlates with a novel subcategory of melanoma cells. Front Immunol. (2023) 14:1196892. doi: 10.3389/fimmu.2023.1196892 37435067 PMC10332463

[30] Yu G Wang LG Han Y He QY. clusterProfiler: an R package for comparing biological themes among gene clusters. OMICS. (2012) 16:284–7. doi: 10.1089/omi.2011.0118 PMC3339379 22455463

[31] Jin S Guerrero-Juarez CF Zhang L Chang I Ramos R Kuan CH. Inference and analysis of cell-cell communication using CellChat. Nat Commun. (2021) 12:1088. doi: 10.1038/s41467-021-21246-9 33597522 PMC7889871

[32] Liu J Lichtenberg T Hoadley KA Poisson LM Lazar AJ Cherniack AD. An integrated TCGA pan-cancer clinical data resource to drive high-quality survival outcome analytics. Cell. (2018) 173:400–416.e11. doi: 10.1016/j.cell.2018.02.052 29625055 PMC6066282

[33] Xu H Yuan Q Wu Z Xu Y Chen J. Integrative transcriptome and single-cell sequencing technology analysis of the potential therapeutic benefits of oleanolic acid in liver injury and liver cancer. Aging (Albany NY). (2023) 15:15267–86. doi: 10.18632/aging.205349 PMC10781501 38127054

[34] Xing J Cai H Lin Z Zhao L Xu H Song Y. Examining the function of macrophage oxidative stress response and immune system in glioblastoma multiforme through analysis of single-cell transcriptomics. Front Immunol. (2024) 14:1288137. doi: 10.3389/fimmu.2023.1288137 38274828 PMC10808540

[35] Li XY Zhao ZJ Wang JB Shao YH Hui-Liu You JX. m7G methylation-related genes as biomarkers for predicting overall survival outcomes for hepatocellular carcinoma. Front Bioeng Biotechnol. (2022) 10:849756. doi: 10.3389/fbioe.2022.849756 35620469 PMC9127183

[36] Wang Y Zhao ZJ Kang XR Bian T Shen ZM Jiang Y. lncRNA DLEU2 acts as a miR-181a sponge to regulate SEPP1 and inhibit skeletal muscle differentiation and regeneration. Aging (Albany NY). (2020) 12:24033–56. doi: 10.18632/aging.104095 PMC7762514 33221762

[37] Chi H Gao X Xia Z Yu W Yin X Pan Y. FAM family gene prediction model reveals heterogeneity, stemness and immune microenvironment of UCEC. Front Mol Biosci. (2023) 10:1200335. doi: 10.3389/fmolb.2023.1200335 37275958 PMC10235772

[38] Liu G Xiong D Che Z Chen H Jin W. A novel inflammation-associated prognostic signature for clear cell renal cell carcinoma. Oncol Lett. (2022) 24:307. doi: 10.3892/ol.2022.13427 35949606 PMC9353224

[39] Newman AM Liu CL Green MR Gentles AJ Feng W Xu Y. Robust enumeration of cell subsets from tissue expression profiles. Nat Methods. (2015) 12:453–7. doi: 10.1038/nmeth.3337 PMC4739640 25822800

[40] Yuan Q Lu X Guo H Sun J Yang M Liu Q. Low-density lipoprotein receptor promotes crosstalk between cell stemness and tumor immune microenvironment in breast cancer: a large data-based multi-omics study. J Transl Med. (2023) 21:871. doi: 10.1186/s12967-023-04699-y 38037058 PMC10691045

[41] Lin Z Fan W Sui X Wang J Zhao J. Necroptosis-related lncRNA signatures for prognostic prediction in uterine corpora endometrial cancer. Reprod Sci. (2023) 30:576–89. doi: 10.1007/s43032-022-01023-9 PMC9988759 35854199

[42] Zhao J Jiao W Sui X Zou J Wang J Lin Z. Construction of a prognostic model of luteolin for endometrial carcinoma. Am J Transl Res. (2023) 15:2122–39. PMC10086912 37056832

[43] Geeleher P Cox N Huang RS. pRRophetic: an R package for prediction of clinical chemotherapeutic response from tumor gene expression levels. PloS One. (2014) 9:e107468. doi: 10.1371/journal.pone.0107468 25229481 PMC4167990

[44] Zou J Lin Z Jiao W Chen J Lin L Zhang F. A multi-omics-based investigation of the prognostic and immunological impact of necroptosis-related mRNA in patients with cervical squamous carcinoma and adenocarcinoma. Sci Rep. (2022) 12:16773. doi: 10.1038/s41598-022-20566-0 36202899 PMC9537508

[45] Zhao J Zou J Jiao W Lin L Wang J Lin Z. Construction of N-7 methylguanine-related mRNA prognostic model in uterine corpus endometrial carcinoma based on multi-omics data and immune-related analysis. Sci Rep. (2022) 12:18813. doi: 10.1038/s41598-022-22879-6 36335189 PMC9637130

[46] Small W Jr Bacon MA Bajaj A Chuang LT Fisher BJ Harkenrider MM. Cervical cancer: A global health crisis. Cancer. (2017) 123:2404–12. doi: 10.1002/cncr.30667 28464289

[47] Brianti P De Flammineis E Mercuri SR. Review of HPV-related diseases and cancers. New Microbiol. (2017) 40:80–5. 28368072

[48] Jin W Yao Q Liu Z Cao W Zhang Y Che Z. Do eye diseases increase the risk of arthritis in the elderly population? Aging (Albany NY). (2021) 13:15580–94. doi: 10.18632/aging.203122 PMC8221314 34111026

[49] Gu X Cai L Luo Z Shi L Peng Z Sun Y. Identification and validation of a muscle failure index to predict prognosis and immunotherapy in lung adenocarcinoma through integrated analysis of bulk and single-cell RNA sequencing data. Front Immunol. (2023) 13:1057088. doi: 10.3389/fimmu.2022.1057088 36733390 PMC9888242

[50] Luo Z He Z Qin H Chen Y Qi B Lin J. Exercise-induced IL-15 acted as a positive prognostic implication and tumor-suppressed role in pan-cancer. Front Pharmacol. (2022) 13:1053137. doi: 10.3389/fphar.2022.1053137 36467072 PMC9712805

[51] Reich O Regauer S. Elimination of reserve cells for prevention of HPV-associated cervical cancer. Virus Res. (2023) 329:199068. doi: 10.1016/j.virusres.2023.199068 36854360 PMC10194256

[52] Chen YH Hueng DY Tsai WC. Proteolipid protein 2 overexpression indicates aggressive tumor behavior and adverse prognosis in human gliomas. Int J Mol Sci. (2018) 19:3353. doi: 10.3390/ijms19113353 30373180 PMC6274732

[53] Sonoda Y Warita M Suzuki T Ozawa H Fukuda Y Funakoshi-Tago M. Proteolipid protein 2 is associated with melanoma metastasis. Oncol Rep. (2010) 23:371–6. doi: 10.3892/or 20043097

[54] Lee SM Shin H Jang SW Shim JJ Song IS Son KN. PLP2/A4 interacts with CCR1 and stimulates migration of CCR1-expressing HOS cells. Biochem Biophys Res Commun. (2004) 324:768–72. doi: 10.1016/j.bbrc.2004.09.118 15474493

[55] Zhou WJ Zhang J Xie F Wu JN Ye JF Wang J. CD45RO-CD8+ T cell-derived exosomes restrict estrogen-driven endometrial cancer development via the ERβ/miR-765/PLP2/Notch axis. Theranostics. (2021) 11:5330–45. doi: 10.7150/thno.58337 PMC8039953 33859750

[56] Zou Y Chen Y Yao S Deng G Liu D Yuan X. MiR-422a weakened breast cancer stem cells properties by targeting PLP2. Cancer Biol Ther. (2018) 19:436–44. doi: 10.1080/15384047.2018.1433497 PMC5915044 29509055

[57] Ding Z Jian S Peng X Liu Y Wang J Zheng L. Loss of miR-664 expression enhances cutaneous Malignant melanoma proliferation by upregulating PLP2. Med (Baltimore). (2015) 94:e1327. doi: 10.1097/MD.0000000000001327 PMC4616445 26287415

[58] Adnani L Dixit R Chen X Balakrishnan A Modi H Touahri Y. Plag1 and Plagl2 have overlapping and distinct functions in telencephalic development. Biol Open. (2018) 7:bio038661. doi: 10.1242/bio.038661 30361413 PMC6262857

[59] Voz ML Agten NS Van de Ven WJ Kas K. PLAG1, the main translocation target in pleomorphic adenoma of the salivary glands, is a positive regulator of IGF-II. Cancer Res. (2000) 60:106–13. 10646861

[60] Voz ML Mathys J Hensen K Pendeville H Van Valckenborgh I Van Huffel C. Microarray screening for target genes of the proto-oncogene PLAG1. Oncogene. (2004) 23:179–91. doi: 10.1038/sj.onc.1207013 14712223

[61] Chng KR Chang CW Tan SK Yang C Hong SZ Sng NY. A transcriptional repressor co-regulatory network governing androgen response in prostate cancers. EMBO J. (2012) 31:2810–23. doi: 10.1038/emboj.2012.112 PMC3380210 22531786

[62] Cheng M Zeng Y Zhang T Xu M Li Z Wu Y. Transcription factor ELF1 activates MEIS1 transcription and then regulates the GFI1/FBW7 axis to promote the development of glioma. Mol Ther Nucleic Acids. (2020) 23:418–30. doi: 10.1016/j.omtn.2020.10.015 PMC7787950 33473327

[63] Blackwood EA Hofmann C Santo Domingo M Bilal AS Sarakki A Stauffer W. ATF6 regulates cardiac hypertrophy by transcriptional induction of the mTORC1 activator, rheb. Circ Res. (2019) 124:79–93. doi: 10.1161/CIRCRESAHA.118.313854 30582446 PMC6461398

[64] Tan JH Cao RC Zhou L Zhou ZT Chen HJ Xu J. ATF6 aggravates acinar cell apoptosis and injury by regulating p53/AIFM2 transcription in Severe Acute Pancreatitis. Theranostics. (2020) 10:8298–314. doi: 10.7150/thno.46934 PMC7381726 32724472

[65] Ho YJ Lin YM Huang YC Yeh KT Lin LI Lu JW. Tissue microarray-based study of hepatocellular carcinoma validating SPIB as potential clinical prognostic marker. Acta Histochem. (2016) 118:38–45. doi: 10.1016/j.acthis.2015.11.005 26610895

[66] Xiao J Huang K Lin H Xia Z Zhang J Li D. Mogroside IIE inhibits digestive enzymes via suppression of interleukin 9/interleukin 9 receptor signalling in acute pancreatitis. Front Pharmacol. (2020) 11:859. doi: 10.3389/fphar.2020.00859 32587518 PMC7298197

[67] Li Z Zhou H Xia Z Xia T Du G Franziska SD. HMGA1 augments palbociclib efficacy via PI3K/mTOR signaling in intrahepatic cholangiocarcinoma. biomark Res. (2023) 11:33. doi: 10.1186/s40364-023-00473-w 36978140 PMC10053751

[68] Soltani M Zhao Y Xia Z Ganjalikhani Hakemi M Bazhin AV. The importance of cellular metabolic pathways in pathogenesis and selective treatments of hematological Malignancies. Front Oncol. (2021) 11:767026. doi: 10.3389/fonc.2021.767026 34868994 PMC8636012

[69] Peña-Romero AC Orenes-Piñero E. Dual effect of immune cells within tumor microenvironment: pro- and anti-tumor effects and their triggers. Cancers (Basel). (2022) 14:1681. doi: 10.3390/cancers14071681 35406451 PMC8996887

[70] Bi KW Wei XG Qin XX Li B. BTK has potential to be a prognostic factor for lung adenocarcinoma and an indicator for tumor microenvironment remodeling: A study based on TCGA data mining. Front Oncol. (2020) 10:424. doi: 10.3389/fonc.2020.00424 32351880 PMC7175916

[71] Aran D Sirota M Butte AJ. Systematic pan-cancer analysis of Tumor purity [published correction appears in Nat Commun. Nat Commun. (2015) 6:8971. doi: 10.1038/ncomms9971 26634437 PMC4671203

[72] Ma R Yi B Riker AI Xi Y. Metformin and cancer immunity. Acta Pharmacol Sin. (2020) 41:1403–9. doi: 10.1038/s41401-020-00508-0 PMC7656961 32868904

[73] Heckman-Stoddard BM DeCensi A Sahasrabuddhe VV Ford LG. Repurposing metformin for the prevention of cancer and cancer recurrence. Diabetologia. (2017) 60:1639–47. doi: 10.1007/s00125-017-4372-6 PMC5709147 28776080

[74] Rozkiewicz D Hermanowicz JM Tankiewicz-Kwedlo A Sieklucka B Pawlak K Czarnomysy R. The intensification of anticancer activity of LFM-A13 by erythropoietin as a possible option for inhibition of breast cancer. J Enzyme Inhib Med Chem. (2020) 35:1697–711. doi: 10.1080/14756366.2020.1818738 PMC7717683 32912025

[75] Higurashi T Hosono K Takahashi H Komiya Y Umezawa S Sakai E. Metformin for chemoprevention of metachronous colorectal adenoma or polyps in post-polypectomy patients without diabetes: a multicentre double-blind, placebo-controlled, randomised phase 3 trial. Lancet Oncol. (2016) 17:475–83. doi: 10.1016/S1470-2045(15)00565-3 26947328

[76] Veneziani I Infante P Ferretti E Melaiu O Battistelli C Lucarini V. Nutlin-3a enhances natural killer cell-mediated killing of neuroblastoma by restoring p53-dependent expression of ligands for NKG2D and DNAM-1 receptors. Cancer Immunol Res. (2021) 9:170–83. doi: 10.1158/2326-6066.CIR-20-0313 33303573

[77] Shaw AT Ou SH Bang YJ Camidge DR Solomon BJ Salgia R. Crizotinib in ROS1-rearranged non-small-cell lung cancer. N Engl J Med. (2014) 371:1963–71. doi: 10.1056/NEJMoa1406766 PMC4264527 25264305

[78] Shaw AT Riely GJ Bang YJ Kim DW Camidge DR Solomon BJ. Crizotinib in ROS1-rearranged advanced non-small-cell lung cancer (NSCLC): updated results, including overall survival, from PROFILE 1001. Ann Oncol. (2019) 30:1121–6. doi: 10.1093/annonc/mdz131 PMC6637370 30980071

[79] Zhong F Wang T Li W Zhang H Zeng X Geisler D. Associations of Single versus Multiple HPV-infection with the Prevalence of Cervical CIN2+ Lesions: HPV type-specific attribution. Lab Invest. (2024) 16:100328. doi: 10.1016/j.labinv.2024.100328 38237737

[80] Li J Cao Y Liu Y Yu L Zhang Z Wang X. Multiomics profiling reveals the benefits of gamma-delta (γδ) T lymphocytes for improving the tumor microenvironment, immunotherapy efficacy and prognosis in cervical cancer. J Immunother Cancer. (2024) 12:e008355. doi: 10.1136/jitc-2023-008355 38199610 PMC10806547

[81] Shapir Itai Y Barboy O Salomon R Bercovich A Xie K Winter E. Bispecific dendritic-T cell engager potentiates anti-tumor immunity. Cell. (2024) 187:375–389.e18. doi: 10.1016/j.cell.2023.12.011 38242085

