# Supplementary figures and images for: Decoding the tumor microenvironment and molecular mechanism: unraveling cervical cancer subpopulations and prognostic signatures through scRNA-Seq and bulk RNA-seq analyses
Source: Front Immunol. 2024 Feb 28;15:1351287. doi: 10.3389/fimmu.2024.1351287 (PMC10933018; doi:10.3389/fimmu.2024.1351287)

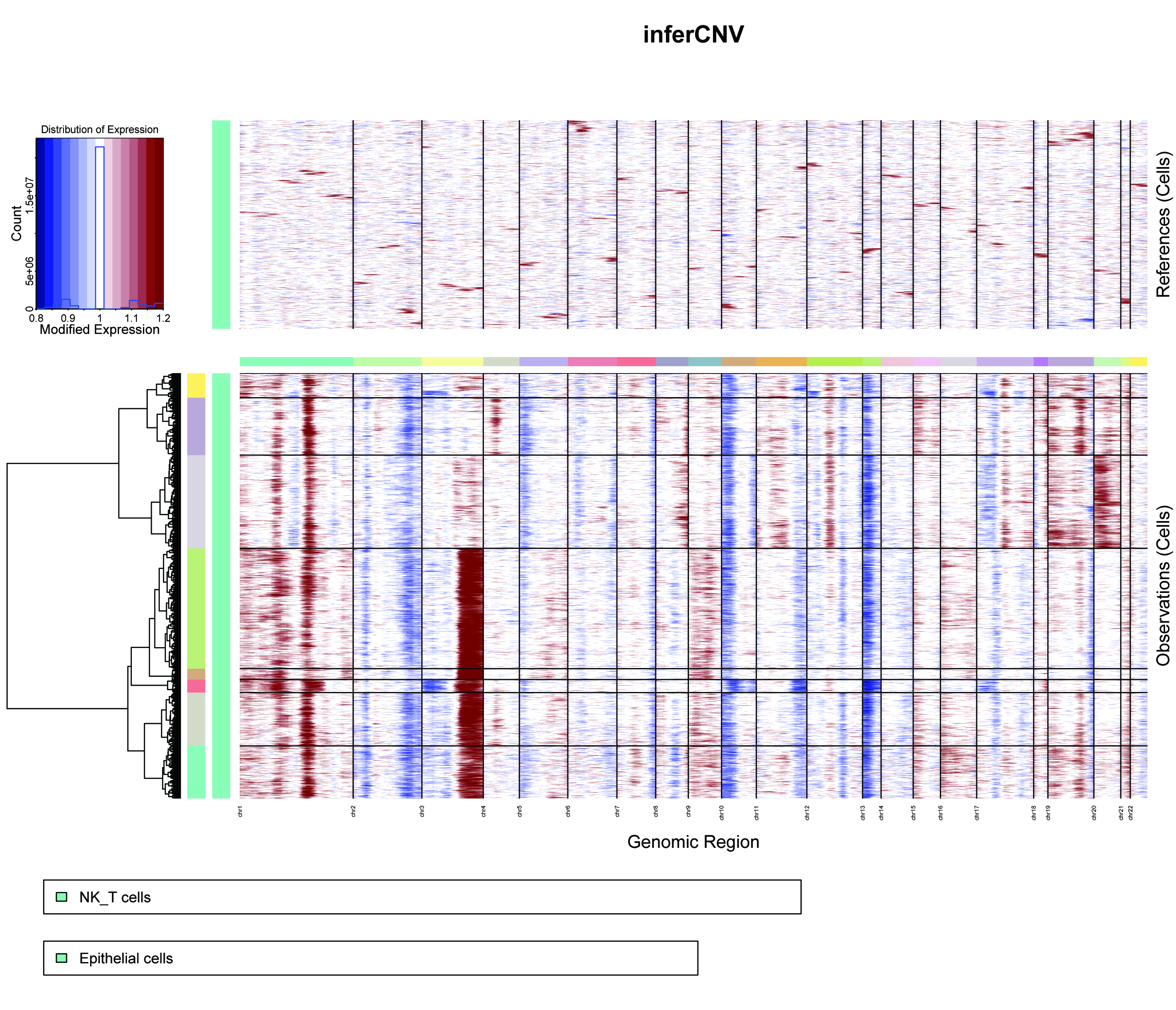

Supplement: Supplementary Figure 1 — The InferCNV heatmap illustrates copy number variations in Epithelial cells, using NK T cells as reference cells. [file Image_1.tif]

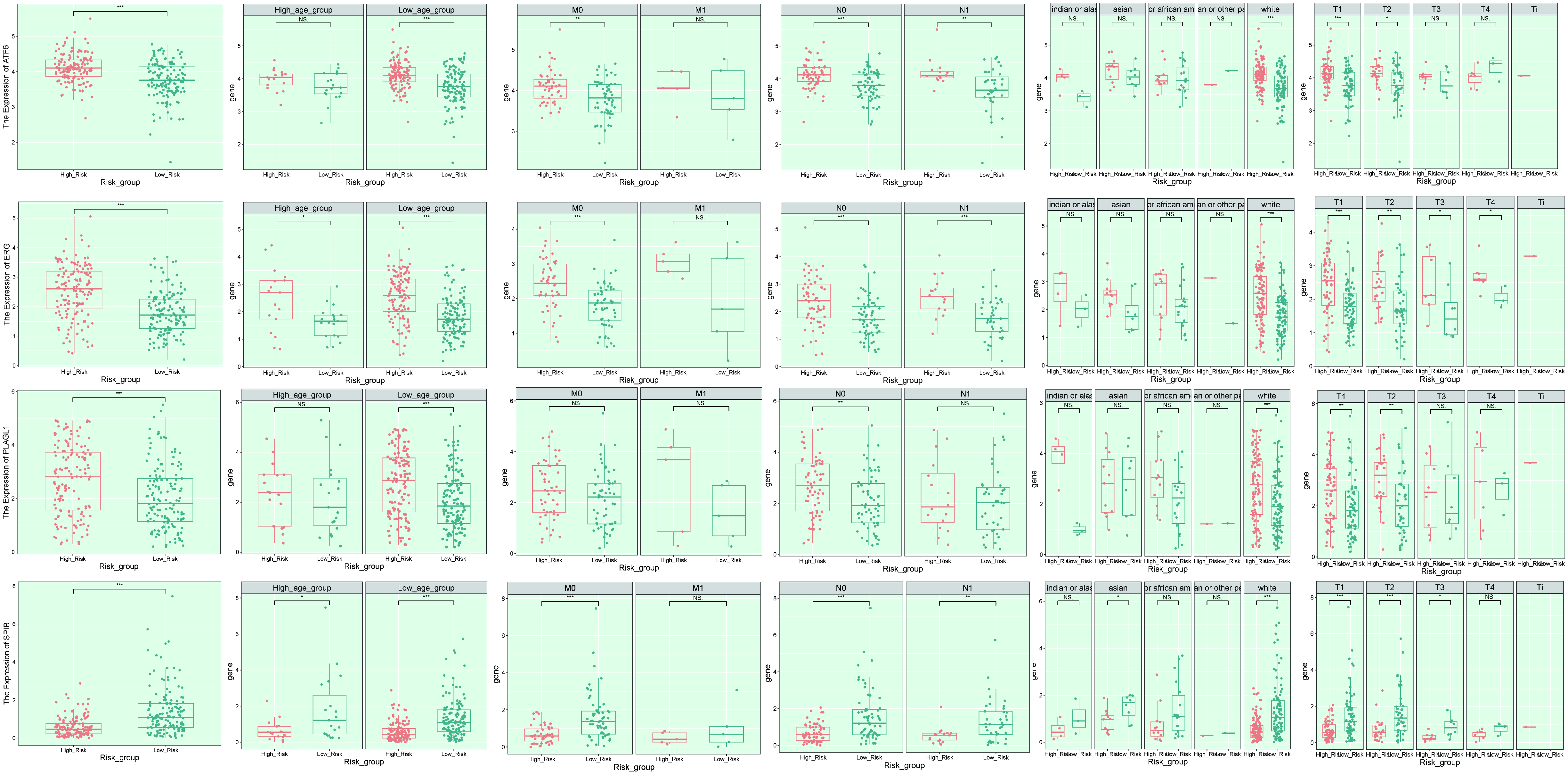

Supplement: Supplementary Figure 2 — The box plots depict the differential expression of modeled genes in the high PLP2+ Tumor EPCs score group and low PLP2+ Tumor EPCs score group, as well as variations in expression across different age groups, T, N, M stages, and ethnicities. [file Image_2.tif]
