# Supplementary material for: Decoding the tumor microenvironment and molecular mechanism: unraveling cervical cancer subpopulations and prognostic signatures through scRNA-Seq and bulk RNA-seq analyses
Source: Front Immunol. 2024 Feb 28;15:1351287. doi: 10.3389/fimmu.2024.1351287 (PMC10933018; doi:10.3389/fimmu.2024.1351287)
Supplement: Supplementary file 3 [file Table_1.docx]

| **Oligonucleotides** | **Nucleotide sequence (5'-3')** |
| --- | --- |
| **siRNA** |  |
| SiRNA-NC | GCUUCGCGCCGUAGUCUUA |
| Si ATF6-1 | GCAGCAACCAATTATCAGTTT |
| Si ATF6-2 | CCCAGAAGTTATCAAGACTTT |
| **Primer** |  |
| GAPDH | GGCCTCCAAGGAGTAAGACC (forward) |
|  | AGGGGAGATTCAGTGTGGTG (reverse) |
| ATF6 | ACCACTAGTAGTATCAGGAACTCA (forward) |
|  | AATGTGTCTCCCCTTCTGCG (reverse) |
|  |  |

**Table S1. Oligonucleotides used in research**
